# Mechanisms and Applications of Conductive Biomaterials in Spinal Cord Injury Repair

**DOI:** 10.34133/bmr.0381

**Published:** 2026-06-11

**Authors:** Bin Zhao, Zhonghan Wang, Tong Yu, Xiangran Cui, Jinbo Zhang, Quezhu Danzeng, Wenjie Wang, Yi Shen, Chenhao Ma, Yaolin Zhao, Jianhang Jiao, Minfei Wu

**Affiliations:** ^1^ Department of Orthopedics, The Second Hospital of Jilin University, Changchun 130041, PR China.; ^2^ Orthopaedic Research Institute of Jilin Province, Changchun 130041, PR China.; ^3^ Department of Orthopedics, Second Affiliated Hospital of Liaoning University of Traditional Chinese Medicine, Shenyang 110167, PR China.

## Abstract

Spinal cord injury (SCI) is a debilitating disorder of the central nervous system and remains a major challenge in neural regeneration and rehabilitation research. Spinal cord stimulation (SCS) has demonstrated notable efficacy in promoting neural repair and functional recovery following SCI. Its mechanisms include enhancing descending pathway conduction through neural plasticity, suppressing inflammation, and stimulating the secretion of neurotrophic factors, thereby creating a permissive microenvironment for axonal regeneration and remyelination. Nevertheless, in cases of complete SCI or extensive structural damage, SCS alone often shows limited therapeutic benefits. Advances in materials science have introduced conductive biomaterials as a promising strategy for SCI repair. These materials can replicate the spinal cord’s electrical microenvironment, fill lesion sites, promote neural stem cell differentiation, guide directional axonal growth, facilitate remyelination, and modulate immune responses to mitigate secondary injury, collectively contributing to neuroprotection and functional recovery. This review systematically summarizes recent progress in the application of SCS and conductive biomaterials for SCI repair, highlights the current limitations of SCS in clinical settings, and provides an in-depth discussion on the mechanisms and translational potential of conductive biomaterials in neural regeneration.

## Introduction

According to the World Health Organization (WHO), more than 15 million people worldwide are currently living with spinal cord injury (SCI), and its incidence continues to increase annually. The distribution of SCI varies across different spinal regions, with cervical injuries being the most prevalent, representing approximately 54.2% of all cases [1]. Following SCI, particularly in cases of complete SCI, multiple systemic dysfunctions often arise, accompanied by a range of severe complications that substantially reduce patients’ quality of life. Clinically, the current management of SCI has established a multimodal framework centered on acute surgical intervention, pharmacological stabilization, and comprehensive rehabilitation, aiming to promote neural repair, functional recovery, and long-term prognosis. Nevertheless, existing therapeutic strategies remain largely insufficient to achieve substantial restoration of neural function.

Against the backdrop of advancing research in SCI repair, SCS has emerged as a promising approach for neural regeneration. Owing to its notable advantages in improving the spinal microenvironment and promoting neural plasticity, SCS has become a major focus of research in the field of neural regeneration [2,3]. Studies have shown that SCS can effectively suppress local inflammatory responses, reduce glial scar formation, and promote the expression and release of various neurotrophic factors, thereby markedly improving the microenvironment of the injury site and creating favorable conditions for axonal regeneration, remyelination, and neural circuit reconstruction [4]. Nonetheless, SCS still faces notable limitations in promoting neural repair after SCI. Current techniques remain insufficient for effectively restoring disrupted neural pathways, and functional recovery, particularly in cases of complete SCI, remains suboptimal [5]. To address this challenge, researchers have recently developed a range of conductive biomaterials that combine excellent electrical conductivity with favorable biocompatibility. These materials not only enhance the reparative capacity of neural tissue but also serve as efficient platforms for electrical signal transmission. When used in conjunction with SCS, they generate synergistic effects, markedly improving repair outcomes following SCI [6–9].

At present, conductive biomaterials have become one of the most active research directions in the field of neural regeneration for SCI repair [10,11]. These materials typically possess good electrical conductivity, biocompatibility, and certain mechanical flexibility, enabling them to establish a stable electroactive microenvironment in vivo. This microenvironment can regulate cell membrane potentials and electrical signal transmission, thereby promoting tissue repair and the reconstruction of neural function. According to their composition and conduction mechanisms, conductive biomaterials can generally be classified into 4 categories: conductive polymers (CPs), carbon-based materials, metal nanoparticle-based materials, and phosphorus-based 2-dimensional (2D) nanomaterials [12]. CPs, such as polypyrrole (PPy), polyaniline (PANI), and polythiophene (PT) derivatives, rely on the π-conjugated structure within their main chains to form delocalized electron systems. During the doping process, charge carriers such as polarons or bipolarons are generated, enabling efficient electron transport. These materials often exhibit both electronic conductivity and a certain degree of ionic conductivity, allowing them to maintain stable electroactivity and favorable interfacial transport properties even in moist physiological environments. Because their mechanical properties are highly tunable, composite biomaterials based on CPs can better match the elastic modulus of soft spinal cord tissue, thereby reducing adverse reactions caused by interfacial mechanical mismatch. In addition, their surface charge states and microstructures can be dynamically regulated through electrochemical approaches, which helps optimize cell–material interactions and effectively promotes neuronal adhesion, axonal extension, and neural network reconstruction [13]. When combined with exogenous electrical stimulation (ES) strategies, CPs can further enhance electrical signal transmission within the injury site, thereby improving overall functional recovery [14]. Carbon-based conductive materials, including graphene, graphene oxide, and carbon nanotubes (CNT), mainly achieve high-mobility electron transport through the continuous π-electron delocalization network formed by sp^2^-hybridized carbon atoms. Their electrical conductivity is markedly higher than that of most organic polymers. These materials also possess large specific surface areas and excellent mechanical strength, enabling the formation of 3D conductive network structures that enhance electrical signal propagation within the injury site. In SCI models, carbon-based materials have been shown to promote neurite outgrowth, enhance synapse formation, and improve the synchronization of neuronal electrical activity. They can also regulate inflammatory responses and glial scar formation to some extent, thereby improving the regenerative microenvironment [15]. Metal nanoparticle-based conductive materials, such as gold, silver, and platinum nanoparticles (PtNP), achieve efficient charge transport through free electrons within the metal structure. These materials exhibit extremely low interfacial impedance and excellent electrochemical stability. They are often used as conductive fillers or electrode modification layers to optimize signal transmission efficiency and interfacial stability in neural ES systems. In SCI repair, different types of metal nanoparticles exhibit distinct biological effects. For example, gold nanoparticles (AuNPs) can promote cell adhesion and neuronal differentiation, thereby facilitating neural functional recovery. PtNPs possess certain antioxidant catalytic activities that can alleviate oxidative stress damage, while silver nanoparticles (AgNPs) exhibit strong antibacterial properties that help reduce the risk of implant-associated infections [12]. These characteristics highlight the considerable potential of metal nanoparticles in SCI repair applications. Phosphorus-based 2D nanomaterials, such as black phosphorus (BP), have attracted increasing attention because of their unique semiconductor properties and biodegradability. These materials possess tunable bandgap structures, and their charge transport relies on migration behavior within layered crystal structures, which also enables excellent photoelectric responsiveness. Unlike many conventional nondegradable conductive biomaterials, some phosphorus-based nanomaterials (e.g., BP) can gradually degrade into phosphate ions in vivo, which may participate in adenosine triphosphate (ATP) synthesis and cellular metabolic processes. This property provides them with high biosafety and biodegradability. In the context of SCI, phosphorus-based materials have been reported to reduce oxidative stress, regulate the inflammatory microenvironment, and promote axonal regeneration, demonstrating unique advantages and translational potential in neural regenerative therapies [16].

Different types of conductive biomaterials construct an electrophysiological platform for regulating the SCI microenvironment through their distinct charge transport mechanisms and biological effects. The design concept of these materials originates from mimicking the electrical signal transmission characteristics of neural tissues, aiming to facilitate neural functional reconstruction by enhancing trans-interface neural signal transmission and regulating local electrical activity [9]. Existing studies have shown that conductive biomaterials can not only markedly improve electrical coupling efficiency and signal transmission between neurons but also provide stable 3D physical support at the structural level. This structural support promotes cell adhesion, migration, and axonal extension, thereby creating favorable conditions for the formation of regenerative neural networks. More importantly, when combined with SCS technology, conductive biomaterials can further amplify the neuroplasticity induced by ES, markedly enhancing neural tissue repair efficiency at the injury site and demonstrating a clear synergistic effect [12].

This review will first outline the pathological processes occurring at different stages following SCI, highlighting the multiple barriers they pose to neural repair. It will then focus on the mechanisms and limitations of SCS in promoting functional recovery and neural regeneration. Building on this, the review systematically summarizes the key targets through which conductive biomaterials modulate the injury microenvironment and facilitate neural repair while also discussing their potential synergistic effects when combined with ES. Finally, it provides perspectives on the future applications of conductive biomaterials in spinal cord repair and their pathways toward clinical translation.

## Pathological Features following SCI

**Fig. 1.**
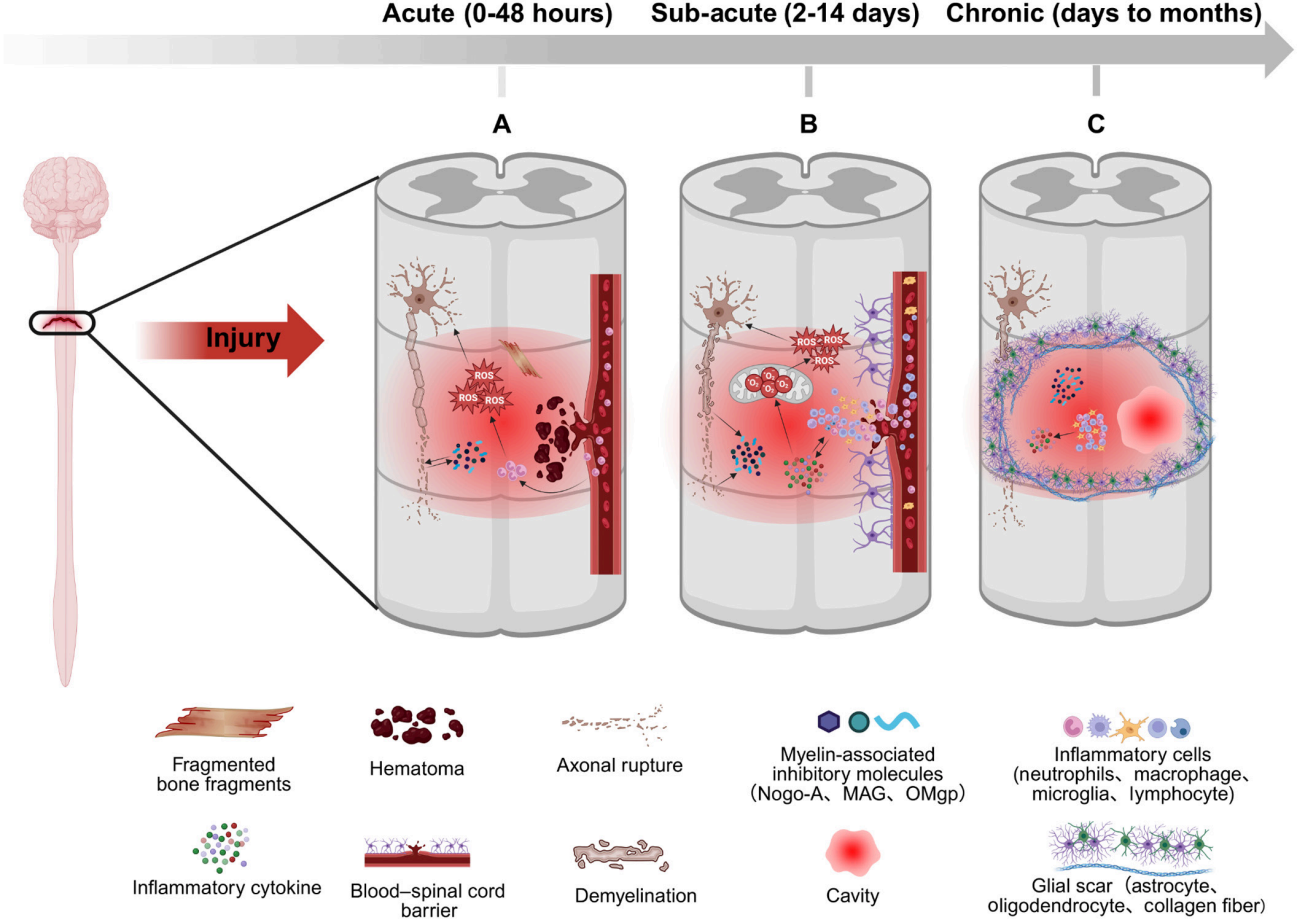
Schematic illustration of the pathological processes at different stages following SCI. (A) Acute phase: External forces and bone fragments directly damage spinal cord structures, resulting in neuronal and vascular injury, axonal disruption, and hematoma formation. During this phase, neutrophils infiltrate the lesion site, secrete inflammatory chemokines, and release reactive oxygen species (ROS) such as superoxide anion (O_2_^−^) and hydrogen peroxide (H_2_O_2_), thereby exacerbating neuronal damage. (B) Subacute phase: Inflammation is markedly enhanced, with extensive infiltration of macrophages and microglia. The released inflammatory factors induce mitochondrial dysfunction and generate large amounts of ROS, further damaging neurons and vascular structures. Meanwhile, disruption of the BSCB allows abnormal infiltration of harmful molecules, aggravating secondary injury and hindering neural repair. (C) Chronic phase: The lesion site develops substantial glial scarring, accompanied by the formation of cystic cavities. Persistent chronic inflammation and impaired remyelination further restrict functional recovery.

Following SCI, depending on the cause and underlying mechanisms, the injury can be classified into primary and secondary damage [17]. Primary injury refers to the instantaneous damage to the spinal cord caused directly by external forces at the moment of trauma. Secondary injury, also known as delayed injury, arises from changes in the local microenvironment following the primary insult, leading to a series of physiological responses and pathological alterations (Fig. 1). Unlike primary injury, secondary injury progresses more gradually but can further exacerbate the severity of SCI [18,19]. Therefore, understanding the differences and underlying mechanisms between primary and secondary injury is essential for the effective treatment and management of patients with SCI.

### Primary injury

Primary injury is mainly caused by direct external forces or other external factors that disrupt the structure and function of spinal cord tissue. Following the injury, the spinal cord may exhibit localized or even complete tissue disruption at the lesion site, directly resulting in neuronal damage [17,19]. In addition, bone fragments resulting from fractures and intervertebral disc displacement caused by vertebral dislocation can exert sustained compression on the spinal cord, further exacerbating neural damage and severely impairing functional recovery [20]. Furthermore, the vasculature surrounding neurons and the neural fibers themselves can also sustain varying degrees of damage due to external forces. Vascular injury or compression can lead to insufficient blood supply to the spinal cord, and the resulting hypoxia and nutrient deprivation may cause cell death, including that of neurons. In addition, hematomas formed from local bleeding following vascular damage can exert persistent compression on spinal cord tissue, further exacerbating SCI [18]. Axonal disruption and myelin damage can result in the loss of neural conduction, leading to motor and sensory deficits below the injury site. Damage to axonal structures and oligodendrocytes can also expose and release myelin-associated inhibitory molecules, such as Nogo-A, MAG, and OMgp, into the injury microenvironment. These myelin-associated inhibitory proteins are intrinsic components of central nervous system (CNS) myelin; however, following SCI, their inhibitory effects are markedly amplified, becoming critical factors that impede neuronal regeneration [21–23]. Regardless of the cause, primary injury immediately induces damage to the spinal cord tissue. This process occurs instantaneously and is considered irreversible, thereby determining the initial severity of SCI [17,19].

Currently, the clinical management of primary injury primarily relies on surgical intervention, which involves the removal of bone fragments, hematomas, and other compressive elements to achieve early decompression of the spinal canal, spinal stabilization, and structural reconstruction. This approach effectively prevents further spinal cord damage and provides favorable conditions for neural recovery. However, existing treatments are largely focused on mechanical decompression and lack effective interventions at the cellular level. Moreover, postoperative progression of secondary injury and incomplete functional recovery remain potential risks [24].

### Secondary injury

Secondary injury, also referred to as delayed or secondary damage, is a pathological cascade directly triggered by the primary insult. It is initiated immediately following the primary injury and can persist for months or even years. The progression of secondary injury can lead to an expansion of the affected SCI region, ultimately resulting in severe neurological dysfunction [25]. Secondary injury essentially comprises a series of complex cellular and biological responses triggered by the primary insult. Damage to neurons, myelin, and surrounding normal tissue structures following the primary injury initiates a cascade of interconnected pathological events, including neuronal apoptosis and necrosis, exacerbated inflammatory responses, disruption of spinal cord hemodynamics, oxidative stress induced by excessive generation of reactive oxygen species (ROS), dysregulated calcium influx, and the formation of fibrotic glial scars [18,26,27]. These pathological processes are mutually reinforcing, not only leading to extensive neuronal loss but also severely compromising the intrinsic repair mechanisms of spinal cord tissue. Ultimately, this results in irreversible spinal cord dysfunction, markedly hindering neural recovery and imposing long-term, profound adverse effects on patients’ motor and sensory functions as well as overall quality of life. Therefore, effective intervention to mitigate secondary injury following SCI is crucial for alleviating associated complications, promoting neural functional recovery, and improving patient prognosis [19,23,28].

Secondary injury is a critical factor exacerbating spinal cord dysfunction. Based on the timing and underlying mechanisms, it can be divided into 3 stages: the acute phase (0 to 48 h), the subacute phase (48 h to 14 d), and the chronic phase (ranging from several days to years) [25,29]. At different stages following injury, the spinal cord exhibits characteristic pathological changes, and the progressive aggravation of these alterations further exacerbates the severity of SCI. During the early phase of secondary injury (acute and subacute phases), structural damage to neural tissue and vascular endothelial cells at the lesion site leads to the release of a large number of cytokines and chemokines. These bioactive molecules further activate local immune responses, recruiting and activating immune cells to establish an inflammatory microenvironment [25,30]. During this stage, microglia and astrocytes are markedly activated. Activated microglia release large amounts of proinflammatory factors, such as tumor necrosis factor-α (TNF-α), interleukin-1β (IL-1β), and IL-6, thereby amplifying the inflammatory cascade and further inducing neuronal necrotic death through mediation of mitochondrial damage [31]. Meanwhile, microglia can also increase vascular permeability by up-regulating factors such as vascular endothelial growth factor (VEGF), thereby compromising the structural integrity of the blood–spinal cord barrier (BSCB). This leads to plasma protein and fluid extravasation, inducing spinal cord edema and further exacerbating neural functional deficits [32]. Simultaneously, excessively activated astrocytes undergo abnormal proliferation, leading to the formation of dense glial scars. Glial scarring plays a dual role in spinal cord repair. In the early stages of injury, it helps contain the spread of inflammation and stabilizes the lesion site, thereby protecting surrounding uninjured tissue. However, over time, this scar tissue acts as a physical barrier that impedes neural repair. Additionally, astrocytes secrete inhibitory molecules such as chondroitin sulfate proteoglycans (CSPGs), which, in combination with myelin-associated inhibitors (e.g., Nogo-A and MAG), create a chemically inhibitory microenvironment that is unfavorable for neural regeneration. This environment markedly restricts axonal regeneration and extension, representing a major obstacle to functional neural recovery [33–35].

During the chronic phase of SCI, a series of relatively stable and persistent pathological features emerge. Microglia and macrophages remain in a state of sustained low-level activation. This chronic activation not only maintains long-term neuroinflammation but also continuously disrupts the homeostasis of the local neural microenvironment through the secretion of proinflammatory factors and ROS [36,37]. Meanwhile, the glial scar becomes stabilized, forming a dense barrier that markedly inhibits neural regeneration. During this phase, cystic cavities are often observed within the lesion site, neuronal apoptosis and axonal disruption gradually reach an irreversible stage, and impaired remyelination following demyelinating lesions further contributes to persistent deficits in neural conduction [38]. Notably, even as the inflammatory response gradually subsides during the chronic phase, residual myelin debris and glial scars persist within the lesion site. Myelin-associated inhibitory proteins, particularly Nogo-A, remain expressed and can activate neuronal Rho/ROCK signaling pathways, continuously suppressing axonal growth and extension, thereby reinforcing the inhibitory microenvironment [39]. These persistently present inhibitory factors constitute a major barrier to neural functional recovery, severely impeding the repair process following SCI.

Current clinical interventions for secondary SCI primarily focus on suppressing neuroinflammation, alleviating oxidative stress, protecting neural cells, and maintaining spinal cord microenvironmental homeostasis. Among these, pharmacological treatment remains the most commonly employed strategy. In the early phase of injury, high-dose glucocorticoids (e.g., methylprednisolone) are often administered to inhibit inflammation and reduce tissue edema. Although their efficacy and safety remain somewhat controversial, their use in the acute phase still holds certain clinical value [40,41]. Mannitol is commonly used to reduce intramedullary pressure, thereby alleviating secondary compressive injury caused by edema or hemorrhage [42,43]. In the chronic phase of injury, neurotrophic agents may be administered to protect neurons [44,45]. Additionally, hyperbaric oxygen therapy and hypothermia, as adjunctive interventions, aim to mitigate secondary injury by improving tissue oxygenation or reducing metabolic demand [46–48]. Rehabilitation training also plays a role in preventing disuse atrophy of muscles below the injury level and promoting neural functional recovery [49].

However, despite the implementation of comprehensive therapeutic strategies, patients with SCI ultimately progress to a relatively stable pathological plateau, characterized by long-term, often irreversible, multisystem functional impairments [50]. Therefore, a thorough understanding of the pathological mechanisms at different stages of SCI and the development of precise, stage-specific interventions are crucial for enhancing repair outcomes and improving patient prognosis. Building on this, emerging approaches such as SCS offer novel strategies to promote neuroplasticity within residual neural circuits and to overcome the inhibitory microenvironment characteristic of the chronic phase, demonstrating promising clinical potential [3,51,52].

## SCS for the Repair of SCI

SCS, as an important neuromodulatory approach, applies exogenous electrical currents to the lesion site and adjacent neural networks, thereby enhancing the excitability and synaptic transmission efficiency of residual neural circuits and reshaping intrinsic spinal electrophysiological activity [53]. In addition, SCS can regulate the expression of brain-derived neurotrophic factor (BDNF) [54], promote axonal regeneration and remyelination, and improve the local inflammatory microenvironment, partially mitigating secondary injury processes. Together, these effects provide electrophysiological and molecular-level support for neural functional recovery after SCI. However, it should be emphasized that SCS alone is essentially a neural function modulation strategy, primarily relying on the regulation and remodeling of residual neural pathways, and lacks the capacity to directly contribute to tissue structural reconstruction [55]. In cases of complete injury or severe tissue loss, ES alone is unable to bridge anatomical discontinuities to establish new neural connections, and it cannot provide the physical scaffolding or spatial guidance necessary for axonal growth. Thus, its ability to repair tissue structure remains limited [56].

Based on this, before discussing strategies for combined use of conductive biomaterials and ES in SCI repair, it is necessary first to systematically describe the mechanisms of action of SCS and analyze its limitations in SCI recovery. Subsequently, the focus will shift to the functional characteristics of conductive biomaterials in SCI repair and their potential to complement SCS in structural reconstruction, thereby facilitating axonal regeneration across the lesion site and promoting functional recovery.

### Mechanisms of SCS in SCI repair

SCS has shown marked potential in promoting neural repair after SCI. Although the precise cellular and molecular mechanisms of ES remain incompletely understood, existing studies indicate that SCS enhances the plasticity of spinal neural circuits, thereby providing a foundation for neuronal survival and axonal regeneration. At the same time, SCS can up-regulate neurotrophic factors, modulate local neuroinflammatory responses, and improve the microenvironment of the injured area, creating favorable conditions for axonal regrowth and remyelination [57,58].

**Fig. 2.**
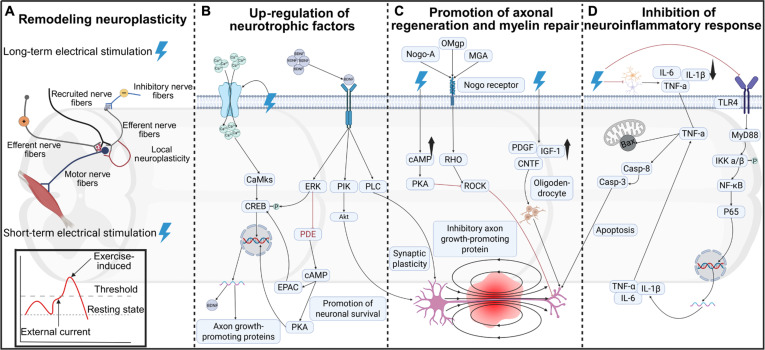
Schematic illustration of the mechanisms by which SCS promotes SCI repair. (A) Mechanisms of long-term SCS in remodeling neural plasticity and short-term SCS in improving neural function. (B) Mechanisms of SCS in up-regulating neurotrophic factors and the role of these factors in neural repair. (C) Mechanisms of SCS in promoting axonal regeneration and myelin repair. (D) Mechanisms of SCS in modulating neuroinflammatory responses.

A growing body of evidence has demonstrated that SCS promotes spinal cord repair and functional recovery through multiple mechanisms, and its therapeutic potential in neuroregeneration continues to be validated. However, the repair mechanisms mediated by SCS are not yet fully clarified, particularly regarding how ES precisely regulates cellular behaviors, signaling pathways, and the local microenvironment. Current evidence suggests that SCS may exert its reparative effects by up-regulating neurotrophic factors, suppressing excessive inflammation, and facilitating axonal regeneration and synaptic remodeling (Fig. 2). These findings provide important clues for understanding the mechanisms of SCS. In the following sections, we will systematically discuss the specific mechanisms by which SCS contributes to SCI repair.

#### Remodeling neural plasticity

Neural plasticity refers to the ability of the nervous system to reconstruct and adapt by modifying neuronal structure and function in response to external stimuli such as SCS. It is a fundamental mechanism of functional recovery and plays a critical role in SCI repair [59]. As early as the 20th century, neuroscience pioneers Charles Sherrington and Thomas Graham Brown revolutionized the understanding of neural control by introducing the concept of central pattern generators (CPGs)—spinal networks capable of independently generating rhythmic movements such as walking and swimming, even in the absence of supraspinal input or peripheral feedback [60]. Early experiments in dogs with complete spinal transection showed involuntary gait-like movements below the lesion. Later, Philippson observed similar phenomena in cats and dogs, concluding that spinal locomotor activity is governed by central and reflex mechanisms, mediated in part by proprioceptive input acting on spinal centers [61,62]. Indirect evidence suggests that CPGs are also preserved in humans. For instance, Calancie et al. [63] reported that patients with chronic cervical SCI displayed involuntary, rhythmical, alternating, and forceful stepping-like leg movements in a supine position, involving nearly all lower-limb muscles. Experimental studies have shown that short-term stimulation can provide baseline excitability to neurons, allowing residual spinal inputs to reach the threshold for functional responses, which may underlie the immediate improvements observed with SCS [64,65]. More importantly, long-term stimulation can induce sustained functional recovery even in the absence of ongoing stimulation (Fig. 2A). Asboth et al. [66] demonstrated that rats receiving electrochemical neuromodulation training regained voluntary movement without epidural stimulation. Clinically, similar findings have been observed in patients with chronic complete SCI, where long-term training combined with epidural stimulation enabled voluntary leg movements and independent standing even without stimulation [59,67]. These lasting effects cannot be explained solely by increased excitability; instead, they suggest that SCS promotes the remodeling of spinal neural plasticity, leading to the formation of functionally active circuits. Long-term stimulation also induces local plasticity among afferent fibers, interneurons, and motor neurons, strengthening synaptic connections and improving muscle recruitment and motor control [68]. Synaptogenesis enhances motor neuron activation from afferent input, supporting reorganization of neural networks favorable to functional recovery [69]. Furthermore, SCS can increase activity in specific pathways, restoring effective signal transmission across injured segments and generating new “neural memories” [68]. Regulation of inhibitory interneurons also plays a crucial role in rehabilitation [55]. In addition, SCS can activate residual descending pathways, enhancing excitability and excitatory neurotransmitter release, thereby allowing supraspinal centers to regain partial control of spinal circuits and motor output [53]. By stimulating large-diameter proprioceptive fibers, SCS further promotes synapse formation between motor neurons and interneurons, strengthening spinal integration and improving muscle contraction and motor control [70]. Plastic changes within CPG elements may thus contribute both to recovery and to certain pathophysiological states associated with impaired or spontaneous locomotor activity [64].

In summary, neural plasticity is a key mechanism by which SCS promotes functional recovery. It enables residual networks to adapt and reorganize in response to stimulation, enhances excitability and synaptic connectivity, and activates CPGs to rebuild locomotor circuits [64,69]. Long-term stimulation induces durable neural reorganization, enabling voluntary motor function even without stimulation [66,67]. By engaging proprioceptive fibers and residual descending pathways, SCS strengthens central control, improves muscle recruitment, and supports motor learning [53,71]. These insights provide a solid foundation for understanding plasticity-driven mechanisms and optimizing therapeutic strategies.

#### Up-regulation of BDNF expression

ES can markedly up-regulate the expression of BDNF in the spinal cord. For example, Ghorbani et al. [72] reported that prolonged epidural SCS (eSCS) markedly increased BDNF levels in T10 spinal cord-injured rats. Wang et al. [73] further investigated the underlying mechanism of BDNF up-regulation following ES and suggested that ES increases BDNF expression in spinal neurons via Ca^2+^- and extracellular signal-regulated kinase (ERK)-dependent signaling pathways (Fig. 2B). Peripheral nerve stimulation also exerts similar effects; in a sciatic nerve injury model, daily ES (20 Hz, 1 h/d) markedly enhanced BDNF expression in spinal motor neurons [74]. BDNF up-regulation plays a critical role in neural repair after SCI. Jin et al. [75] demonstrated that transplantation of fibroblasts genetically modified to overexpress BDNF (Fb/BDNF) into adult rats with complete unilateral hemisection cervical SCI promoted axonal regeneration. Four to 5 weeks post-transplantation, anterograde tracing with biotinylated dextran amine (BDA) revealed that the absolute growth distance of the reticulospinal tract (ReST) in the Fb/BDNF group was 1.8-fold greater than in the unmodified fibroblast (Fb/UM) group, whereas axonal growth of the rubrospinal tract (RST) and vestibulospinal tract (VST) increased approximately 4-fold. Functional assessments also showed partial forelimb improvement in the Fb/BDNF group, indicating that BDNF-secreting fibroblasts can markedly promote axonal regeneration of supraspinal motor neurons and support partial functional recovery after chronic SCI. Moreover, reviews have suggested that BDNF up-regulation may facilitate repair through multiple signaling pathways, representing a key mechanism driving post-injury motor recovery [4]. BDNF primarily exerts its effects via the TrkB receptor. Al-Majed et al. [74] reported that brief ES (20 Hz, 1 h/d) after femoral nerve transection markedly increased BDNF and TrkB expression in regenerating motor neurons. The BDNF/TrkB cascade activates 3 major pro-neuronal signaling pathways: the phosphoinositide 3-kinase (PI3K)/Akt pathway, phospholipase C (PLC)-related signaling, and the ERK pathway [76]. PI3K-Akt activation promotes neuronal survival; PLC signaling mediates Ca^2+^ transients, influencing synaptic plasticity; and ERK activation inhibits phosphodiesterase-4 (PDE4), thereby reducing adenosine 3′,5′-monophosphate (cAMP) degradation [77]. Consequently, BDNF up-regulation indirectly elevates intracellular cAMP levels, a second messenger that facilitates axonal regeneration and neural plasticity remodeling [78,79]. Experimental evidence further demonstrates that cAMP modulates multiple downstream signaling cascades, including activation of protein kinase A (PKA), which drives transcriptional programs promoting axon growth [80,81]. Hannila and Filbin [78] also confirmed that increased cAMP expression counteracts the inhibitory effects of myelin-associated proteins such as Nogo-A, MAG, and OMgp on axon growth.

Overall, ES-mediated regulation of neurotrophic factor expression contributes to SCI repair through multiple mechanisms. BDNF directly supports neuronal survival and axonal regeneration, while its TrkB-mediated activation of PI3K/Akt, PLCγ, and ERK pathways cooperatively regulates neuron survival, synaptic remodeling, and functional recovery [74]. Additionally, BDNF-driven activation of the cAMP–PKA–pCREB signaling cascade enhances axon growth potential and mitigates the inhibitory effects of myelin-associated proteins, creating a permissive microenvironment for axonal regeneration [78,80]. These findings provide a theoretical and experimental basis for developing combined therapeutic strategies targeting the BDNF signaling pathway.

#### Promotion of axonal regeneration and myelin repair

After SCI, axonal disruption and myelin degradation directly compromise the integrity of neural conduction pathways, leading to interruption of electrical signal transmission. These pathological changes manifest clinically as sensory deficits, motor dysfunction, and autonomic dysregulation below the injury level. Therefore, promoting effective axonal regeneration and precise myelin repair is crucial for reconstructing neural conduction pathways and restoring impaired neurological function. Evidence indicates that SCS plays an active role in this process (Fig. 2C). Bacova et al. [82] applied oscillating field stimulation and observed abundant nerve fibers and myelin in segments adjacent to the lesion, accompanied by markedly increased expression of neurofilament light chain (NF-L) and myelin basic protein (MBP), supporting the role of ES in myelin repair. Angelin et al. [83] employed epidural electrical stimulation (EES; 30 Hz, 500-μs pulses, 40 min/d) in a rat SCI model. After 5 weeks of stimulation, histological analyses showed that axonal structures were more intact and myelination was markedly enhanced in the stimulated group, suggesting that ES accelerates nerve fiber regeneration and reconstruction, effectively improving neural conduction within the SCI region. Li et al. investigated the effects of spinal EES (50 Hz, 200-μs pulses, 0.045 mA) on oligodendrocytes and myelin repair in a T10 contusion SCI rat model. SCS significantly increased MBP expression, and Luxol fast blue (LFB)-positive areas were markedly enlarged, indicating more complete myelin structures. Additionally, both mRNA and protein levels of 2′,3′-cyclic nucleotide 3′-phosphodiesterase (CNPase), an oligodendrocyte-specific enzyme, were markedly elevated in the ES group, further supporting the positive effects of ES on oligodendrocyte function and myelin formation [84].

Directional guidance also represents an important mechanism by which ES promotes axonal regeneration. Matter et al. [85] demonstrated that periodic reversal of electrode polarity established a stable electric field gradient at the injury site, guiding motor axons toward the cathode and sensory axons toward the anode, thereby promoting orderly axonal extension along spinal pathways. Moreover, ES can alter cell membrane potentials, elevate intracellular cAMP and Ca^2+^ levels, and activate downstream cAMP response element-binding protein (CREB), promoting the expression of axon growth-related genes [86]. Related studies also suggest that SCS can inhibit suppressive signaling pathways, such as BMP4–Smad, mitigating their negative effects on neuronal and oligodendrocyte differentiation, thereby facilitating neural regeneration and myelin repair. By up-regulating neurotrophic factor expression and suppressing neuroinflammation, ES improves the spinal microenvironment, further supporting axonal regeneration and myelin restoration [84,87].

#### Suppression of neuroinflammatory responses

Following SCI, the lesion site triggers a robust inflammatory response, which further exacerbates the progression of secondary injury. In this process, abnormal activation of microglia and astrocytes plays a pivotal role by releasing large amounts of inflammatory mediators, amplifying and spreading neuroinflammation [88,89]. Evidence indicates that SCS exerts a key modulatory effect on neuroinflammation after SCI. Through multi-target and multi-pathway mechanisms, SCS can effectively suppress neuroinflammatory responses, thereby improving the local microenvironment and creating favorable conditions for the recovery of spinal cord function (Fig. 2D) [90,91]. At the molecular level, although heterogeneity exists among studies regarding the regulation of pro-inflammatory factor expression by SCS, current evidence generally supports an anti-inflammatory trend of SCS in SCI repair. A recent systematic review indicated that, under most experimental conditions, SCS markedly inhibits pro-inflammatory factor release while promoting the secretion of anti-inflammatory mediators [92]. Multiple preclinical and clinical studies have confirmed that SCS reduces levels of IL-6 and IL-1β while enhancing expression of the anti-inflammatory cytokine IL-10 [93]. Notably, high-frequency SCS (10 kHz) demonstrates pronounced immunomodulatory effects in clinical applications. For instance, in a patient treated with high-frequency SCS, peripheral blood IL-6 and IL-1β levels were markedly decreased, whereas IL-10 was markedly increased [94]. Collectively, these findings suggest that SCS reshapes the local immune microenvironment by establishing a new pro-/anti-inflammatory balance—down-regulating pro-inflammatory factors while up-regulating anti-inflammatory mediators such as IL-10—thereby alleviating secondary inflammatory responses and associated neural dysfunction after SCI [95]. At the cellular level, Sun et al. [96] demonstrated in rats that SCS suppresses microglial activation by inhibiting colony-stimulating factor-1 (CSF-1) released from dorsal root ganglia (DRG). Similarly, Cedeño et al. [97] observed that conventional low-frequency SCS (4 to 60 Hz, 6 h/d) reduced expression of microglia-specific marker OX-42 and astrocyte-specific marker glial fibrillary acidic protein (GFAP), indicating that SCS attenuates glial overactivation to mitigate neuroinflammation. Hahm et al. [98] further confirmed that daily 30-min bilateral high-frequency transcutaneous SCS (tSCS) for 5 weeks after T12 contusion markedly reduced microglial activation in both dorsal and ventral gray matter regions. Peripheral nerve stimulation also exhibits potential in modulating spinal neuroinflammation. Direct current (DC) ES can regulate macrophage polarization, promoting a shift from the pro-inflammatory M1 phenotype to the anti-inflammatory M2 phenotype, thereby facilitating tissue repair and regeneration. In THP-1-derived macrophage models, DC ES up-regulated typical M2-associated genes (e.g., IL-10 and CD206) in M0 and M1 macrophages while suppressing M1 marker CD86 expression and reducing secretion of pro-inflammatory cytokines IL-1β and IL-6. In contrast, M2 macrophages were less responsive to ES, suggesting that DC ES alleviates inflammation and promotes tissue regeneration primarily through M2 macrophage polarization [99]. Additional mechanisms have also been proposed. Evidence suggests that SCS may suppress the Toll-like receptor 4 (TLR4)/nuclear factor κB (NF-κB) pathway to reduce related inflammatory responses. Moreover, specific SCS parameters, such as polarity and frequency modulation, can influence phosphorylation of ERK and P38 in the mitogen-activated protein kinase (MAPK) family, further modulating inflammatory signaling. Although direct evidence in SCI is limited, the anti-inflammatory effects of SCS observed in other neuropathological conditions indicate that inhibition of NF-κB and MAPK pathways may represent key underlying mechanisms [100].

### Limitations and challenges of SCS in SCI repair

Based on current studies, SCS has demonstrated beneficial effects in promoting neuroplasticity, up-regulating BDNF expression, facilitating axonal regeneration and remyelination, and modulating neuroinflammatory responses. However, its therapeutic efficacy primarily depends on the functional regulation of residual neural network excitability and associated molecular signaling pathways. As a result, it still exhibits certain limitations in terms of structural reconstruction of damaged tissue. Specifically, these limitations can be summarized in 3 aspects. First, SCS mainly exerts its effects by enhancing the excitability of existing neural circuits and synaptic plasticity. In cases where spinal cord tissue is severely disrupted or cavitation has formed within the lesion area, SCS alone is insufficient to induce axonal growth across the injury site to restore anatomical continuity. Second, the spatial distribution of the electric field generated by SCS can be strongly influenced by glial scar formation and cavity structures within the injured tissue, which limits the spatial precision of its regulatory effects on specific cell populations. Third, SCS alone cannot provide a physical scaffold to support cell adhesion and axonal extension, nor does it serve as a carrier for the delivery of bioactive factors. Consequently, its ability to continuously improve the local microenvironment after injury remains limited.

Overall, the limitations of SCS alone in SCI repair can be summarized in 2 main aspects. One is the insufficient support for structural reconstruction of the lesion area and the regeneration-supportive microenvironment. The other is the limited spatial precision of ES and its restricted ability to guide axonal regeneration directionally. Based on these considerations, the following sections will provide a systematic discussion focusing on these 2 aspects.

#### Insufficient structural reconstruction and regenerative microenvironmental support

SCS has shown certain potential in promoting functional recovery after SCI. However, its mechanism of action mainly relies on functional modulation of residual neural circuits, and it still exhibits clear limitations in terms of structural reconstruction of injured tissues. SCS regulates the excitability of spinal neural networks through externally applied electric fields, thereby enhancing signal transmission through residual neural pathways and, to some extent, improving motor and sensory functions below the level of injury. Current studies generally regard SCS as a neuromodulatory strategy, whose primary role is to activate or strengthen neural pathways that remain anatomically intact but functionally suppressed after injury, rather than directly reconstructing damaged anatomical structures [101]. In severe SCI, both primary trauma and subsequent secondary injury responses often result in substantial tissue loss, cavity formation, and interruption of axonal pathways. The lesion area typically lacks a continuous extracellular matrix (ECM) framework, making it difficult for regenerating axons to bridge the injury gap and form stable neural connections. Under such conditions, even if SCS enhances the excitability of spinal neural networks and promotes functional remodeling of residual neural circuits, the resulting recovery is largely manifested as functional reorganization of local neural networks rather than true anatomical reconnection across the injury site. Existing studies have also suggested that although some animal experiments have reported associations between SCS and axonal preservation or limited regeneration, the overall evidence remains limited. Its effects are more commonly reflected in promoting functional recovery of residual tissue or exerting neuroprotective actions, rather than directly inducing large-scale structural regeneration or tissue reconstruction [102]. Therefore, from the perspective of tissue structural repair, the primary advantage of SCS in SCI treatment lies in its ability to regulate neural network function and promote neuroplasticity, whereas its capacity to reconstruct anatomical continuity across the lesion site remains relatively limited.

In addition, although SCS can modulate inflammatory responses and related cellular signaling pathways following SCI to a certain extent, it cannot itself provide the structural support or stable regulatory platform necessary for neural tissue regeneration. Within the lesion region, SCS cannot form 3D scaffold structures, provide cell-adhesive substrates similar to the ECM, or function as a carrier for the delivery of cells or bioactive molecules. Consequently, it is difficult for SCS alone to provide sustained physical support and biological regulatory conditions for cell migration, axonal regeneration, and tissue integration [101]. Due to the lack of a structural basis for maintaining a regenerative microenvironment, the influence of SCS on key regulatory factors within the injury microenvironment, such as neuroinflammatory responses and ECM remodeling, is often indirect or transient, making it difficult to establish a stable and long-term regenerative environment. In severe SCI, the injury region is frequently accompanied by marked tissue loss and cavitation, and the absence of a physical scaffold further restricts regenerating axons from crossing the lesion gap and re-establishing effective neural connections. In contrast, studies in tissue engineering have demonstrated that biomaterial scaffolds can not only provide essential structural support for regenerating axons but also serve as delivery platforms for cells or bioactive factors, thereby promoting neural tissue repair and functional recovery [103]. Therefore, relying solely on SCS is generally insufficient to achieve structural repair of the lesion site, highlighting the necessity of integrating biomaterials or tissue engineering strategies capable of providing structural support and regulating the regenerative microenvironment in SCI therapy to further enhance functional recovery after injury.

#### Limited spatial precision and directional guidance of ES

The therapeutic efficacy of SCS is largely influenced by the structural heterogeneity of injured spinal cord tissue. Pathological changes following SCI, such as glial scar formation, cavity development, and disruption of the ECM, can markedly alter the local electrical conductivity and resistance of the tissue. These changes, in turn, affect the propagation pathways and intensity distribution of externally applied electric fields within the lesion area. Studies have shown that the distribution of the electric field generated by ES in the spinal cord is highly dependent on tissue structure and its conductive properties. Differences in conductivity among various tissue layers can lead to substantial variations in current density and electric field strength, thereby influencing the pattern of signal propagation within spinal cord tissue [104]. Furthermore, computational modeling studies have indicated that the morphological alterations and structural disruption caused by SCI can markedly change the spatial distribution of electric fields within the injured region. This results in heterogeneous variations in both field strength and direction, which may ultimately affect the effective range and therapeutic outcomes of ES [105]. Such structural alterations may act as a “structural barrier” that weakens the regulatory effects of ES on deeper neurons or specific cellular populations. In many cases, the electric field tends to concentrate in local tissue regions near the electrodes, making it difficult to achieve uniform stimulation coverage across the entire lesion site [106].

Consequently, within the complex microenvironment of SCI, SCS often lacks sufficient spatial selectivity in regulating different cell types, including neurons, oligodendrocytes, and astrocytes. It is also difficult for SCS alone to establish electric fields or molecular gradients with clear directionality within the lesion area to guide the oriented growth of regenerating axons. Meanwhile, previous studies have demonstrated that gradient distributions of neurotrophic factors play a crucial role in inducing directional axonal growth and facilitating axonal regeneration across the injury site. Although ES can up-regulate the expression of BDNF, this effect is highly dependent on stimulation parameters such as frequency, intensity, and duration, as well as the timing of stimulation and the injury model used [73]. As a result, the expression levels of neurotrophic factors often vary considerably under different experimental conditions, and their changes are typically transient or fluctuating in nature. In addition, ES lacks precise control over the spatial distribution of neurotrophic factors and therefore cannot readily establish stable molecular gradients with directional guidance within the injury region. This limitation may reduce its ability to effectively promote oriented axonal regeneration. Taken together, although SCS can provide a supportive basis for neural regeneration by enhancing neuronal survival and neural network activity, its ability to guide axons across the inhibitory lesion core and achieve directed regeneration remains limited [107,108]. Therefore, within the complex microenvironment of SCI, SCS primarily functions as a regulator of local neural network activity rather than a strategy capable of achieving precise cellular targeting or reconstructing neural structures across the lesion site.

### The role of conductive biomaterials in addressing the limitations of SCS

The limitations associated with SCS alone during SCI repair partially restrict the full therapeutic potential of this strategy for neural regeneration. In this context, conductive biomaterials with both favorable electrical conductivity and biocompatibility have gradually attracted increasing attention and are considered an important complement to SCS-based therapies. Such materials can not only form 3D biomimetic scaffolds within the lesion site to provide structural support for regenerating axons and migrating cells but also serve as platforms for electrical signal conduction and biological regulation. This dual function helps improve the transmission efficiency and stability of externally applied ES within the injured microenvironment. By providing 3D structural support, establishing a regeneration-supportive microenvironment, and mimicking the electrical conduction characteristics of spinal cord tissue to enhance the effects of ES, conductive biomaterials may help overcome the limitations of SCS alone in both tissue structural reconstruction and electrophysiological regulation. Based on these considerations, the following section will further analyze the potential mechanisms through which conductive biomaterials may compensate for the limitations of SCS in SCI repair.

#### Providing 3D structural support and establishing a regenerative microenvironment

Conductive biomaterials can provide essential structural support to the injured region in SCI by forming 3D porous scaffold structures. This is particularly important in complex pathological microenvironments where severe cavitation or dense glial scar formation occurs at the injury site. Implantation of biocompatible 3D scaffolds into the lesion area can effectively fill injury cavities and maintain structural continuity of the tissue, thereby providing a stable physical framework for regenerating tissues [109,110]. At the same time, such scaffold materials are often able to mimic the structural characteristics and mechanical properties of the natural ECM, creating a favorable microenvironment for cell adhesion, migration, and axonal regeneration [103,107]. Studies have shown that neural tissue engineering scaffolds with elasticity, pore architecture, and microtopological features similar to those of the native neural ECM can markedly promote neuronal growth and axonal extension, thereby facilitating the formation of regenerative neural networks [111].

Further studies have demonstrated that biomaterial scaffolds with interconnected porous structures or oriented microchannels can provide clear physical guidance pathways for regenerating axons, thereby facilitating their growth across the injury site and the re-establishment of neural connections [11]. For example, porous scaffolds with anisotropic channel structures can guide regenerating axons to extend along specific directions, which improves the efficiency of axonal bridging across the lesion and promotes neural pathway reconstruction [110]. In addition, 3D biomaterial scaffolds can serve as delivery vehicles for cells and bioactive factors, further improving the regenerative microenvironment at the injury site. By incorporating biological components such as neural stem cells (NSCs), mesenchymal stem cells, or neurotrophic factors, scaffold materials can promote the differentiation of NSCs into neurons, induce oligodendrocyte formation, facilitate endothelial cell migration, and modulate neuroinflammatory responses. These combined effects support remyelination while also promoting vascular reconstruction and tissue repair [112]. At the same time, studies have confirmed that conductive composite scaffolds not only provide structural support but also enhance electrical coupling between neural cells, thereby promoting neuronal differentiation, axonal extension, and neural network reconstruction [113]. Furthermore, when conductive scaffolds are combined with exogenous ES, they can further enhance neurite outgrowth and facilitate the formation of functional neural connections [114,115].

By constructing 3D conductive scaffolds that integrate both structural support and bioactive regulatory functions, it becomes possible not only to provide structural continuity and regenerative pathways for the injured spinal cord at the tissue level but also to promote neural regeneration through modulation of the local regenerative microenvironment. In this way, conductive biomaterials can partially compensate for the limitations of SCS alone in providing structural support and establishing a regenerative microenvironment, thereby creating more favorable conditions for neural functional reconstruction.

#### Mimicking the spinal cord’s electrical conduction microenvironment to enhance ES effects

The excellent electrical conductivity of conductive biomaterials can significantly improve the transmission and distribution of exogenous electrical signals within the injured region, thereby acting synergistically with SCS to further enhance the regulation of neuroplasticity and functional recovery. First, conductive scaffolds form continuous conductive networks that reduce the equivalent resistance of the lesion site and increase the efficiency of ES within the injured tissue. This, in turn, strengthens the modulation of neuronal membrane potentials and neural network excitability. Studies have shown that conductive hydrogels or composite scaffolds can restore electrical signal transmission along damaged neural pathways, promote neuronal differentiation and axonal extension, and accelerate neural circuit reconstruction [116,117]. Second, by controlling the scaffold’s microstructure and anisotropic conductive properties, conductive scaffolds can optimize the spatial distribution of electric fields within the lesion. For example, scaffolds with aligned fiber structures or oriented microchannels can generate synergistic effects between structural guidance and electrical cues, directing electrical signals along the axis of regenerating axons. This promotes oriented axonal growth and improves the efficiency of neural connection reconstruction.

In addition, 3D biomimetic conductive scaffolds enhance cell adhesion, migration, and differentiation, providing a stable cellular and tissue basis for ES-induced neuroplasticity. These materials can also modulate inflammation, oxidative stress, and angiogenesis to improve the regenerative microenvironment, thereby increasing the sensitivity of neural tissue to ES. For instance, recent studies have demonstrated that conductive hydrogels under ES can suppress inflammatory responses, promote M2 macrophage polarization, and enhance NSC differentiation into neurons, collectively facilitating neural network reconstruction and motor function recovery [118].

From a broader electrophysiological perspective, the core significance of conductive biomaterials in SCI repair lies in their ability to mimic the spinal cord’s electrical conduction environment at the tissue scale. In healthy spinal cord tissue, the white matter axon bundles form low-resistance longitudinal pathways, maintaining relatively ordered current propagation. After SCI, cavitation and glial scar formation in the lesion core substantially increase local resistance, causing dispersed and uneven current paths, which disrupt electric field gradients and weaken the modulation of deep neurons by exogenous stimulation [14,119]. Conductive biomaterials, by forming continuous 3D conductive networks, reduce the effective resistance of the lesion and enable current conduction across interrupted tissue, thereby restoring a relatively stable electric field distribution at the macroscopic level [117]. Moreover, scaffolds with designed microstructures, such as aligned fibers or layered networks, can create a degree of anisotropic conductivity, passively guiding current density via impedance differences and optimizing local electric field gradients and spatial uniformity [116]. This effect essentially represents electric field reconstruction and impedance matching rather than active signal generation. The capacitive coupling between the scaffold and tissue further buffers transient current fluctuations, stabilizing membrane potential modulation and enhancing the consistency of neuronal and glial responses to stimulation. Through the combined mechanisms of conductive pathway reconstruction, electric field spatial optimization, and interface electrophysiological modulation, conductive biomaterials can replicate the spinal cord’s electrical conduction microenvironment at the tissue scale and significantly enhance the biological effects of exogenous ES. This provides a critical physical and biological foundation for neural network reconstruction and functional recovery after SCI.

In summary, conductive biomaterials show great potential for addressing the limitations of SCS in SCI therapy, particularly its restricted ability for structural reconstruction and insufficient electrophysiological regulation. On one hand, these materials can form biomimetic 3D scaffolds that provide physical support for injured spinal cord tissue and create a regenerative microenvironment conducive to axonal growth and cell migration. On the other hand, their excellent electrical conductivity allows them to mimic the spinal cord’s electrical conduction microenvironment, enhancing the transmission efficiency and biological effects of exogenous ES. Accordingly, systematically reviewing the structural composition, conductive properties, and mechanisms of action of different types of conductive biomaterials is of great importance for understanding their potential applications in SCI therapy.

## Conductive Biomaterial-Based Strategies for SCI Repair

**Fig. 3.**
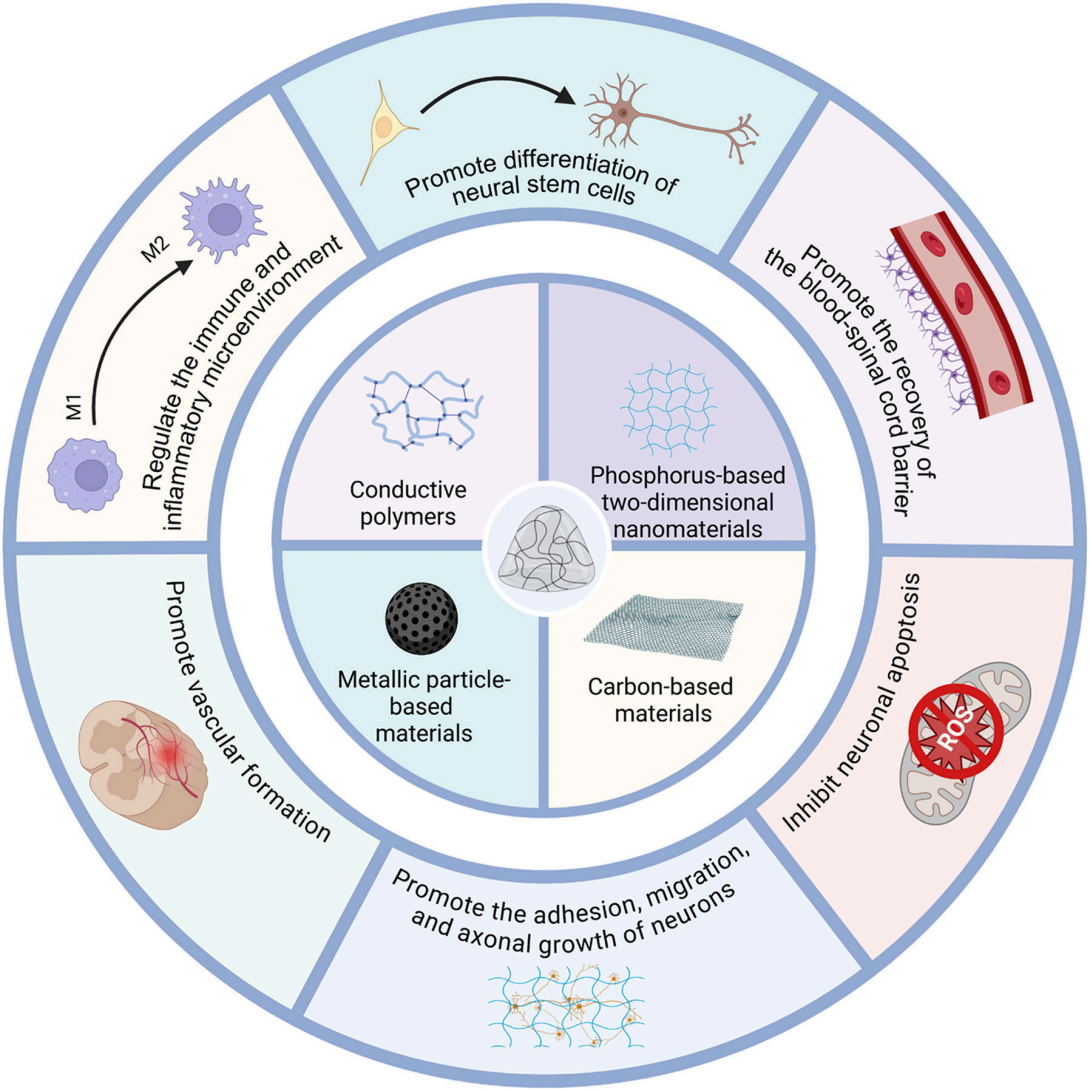
Schematic illustration of the main types of conductive components in conductive biomaterials and their mechanisms in promoting neural regeneration.

Different types of conductive biomaterials exhibit marked variations in electrical conductivity, mechanical properties, biocompatibility, and designable microstructures, and these characteristics largely determine their suitability and potential modes of action in SCI repair. Based on this, we first provide a systematic classification and overview of the main types of conductive biomaterials currently applied in SCI repair, with a focus on analyzing their key physicochemical properties, therapeutic effects, and applicable scenarios. At the same time, the limitations of each material in practical applications are summarized. On this basis, we further discuss the potential mechanisms by which conductive biomaterials can synergize with ES to mediate SCI repair (Fig. 3), providing a theoretical foundation for developing neural repair strategies that integrate functional materials with ES.

### Types of conductive biomaterials

Conductive biomaterials for SCI repair must not only provide electrical conductivity but also exhibit good flexibility, biocompatibility, biodegradability, and the ability to promote neural regeneration. Single-component materials often cannot meet all of these requirements simultaneously. Therefore, conductive biomaterials used in SCI repair are generally multi-component composites. Commonly, these composites consist of a conductive component combined with a matrix material, and in many cases are further supplemented with neurotrophic factors, NSCs, or pharmacological agents to create multifunctional composites capable of synergistically promoting SCI repair [12]. The conductive components of these biomaterials are diverse, each offering specific advantages and suitable applications. In the context of SCI repair, conductive components are generally classified into 4 major categories: (a) CPs, (b) carbon-based materials, (c) metal nanoparticle-based materials, and (d) phosphorus-based 2D nanomaterials [120]. Based on this, the following section systematically classifies and summarizes the types of conductive components currently used in conductive biomaterials for SCI repair. It also provides a comprehensive comparison of CPs, carbon-based materials, metal nanoparticles, and phosphorus-based 2D nanomaterials across multiple dimensions, including electrical conductivity, biocompatibility, biodegradability, applicable stages of injury, and intrinsic material limitations (Table 1). This comparison aims to present a clear overview of the performance characteristics, advantages, and limitations of different classes of conductive biomaterials.

**Table 1.** Comparison of major conductive biomaterials used for SCI repair (references listed in main text). Injury stages: A (acute injury stage); B (subacute injury stage); C (chronic injury stage).

Material type	Representative materials	Electrical conductivity	Biocompatibility	Biodegradability	Mechanical compatibility	Main therapeutic effects	Applicable injury stages	Major limitations
Conductive polymers (CPs)	PPy, PANI, PEDOT, PT	Moderate (~10^−3^–10^2^ S/cm, tunable via doping)	High biocompatibility;markedly improved when combined with natural polymers	Partially biodegradable through composite design	Tunable; better matched to spinal cord tissue when incorporated into hydrogels or biodegradable polymers	Improves electrical signal transmission, modulates inflammation, promotes neural differentiation and axonal regeneration	A, B, C	Potential toxicity from dopants, long-term electrical stability, relatively complex fabrication
Carbon-based materials	Graphene, graphene oxide (GO), carbon nanotubes (CNTs)	Very high (10^2^–10^5^ S/cm)	Moderate; often requires surface functionalization	Limited biodegradability; typically nonbiodegradable	High mechanical strength but limited softness compared to neural tissue	Enhances electrical signal conduction, promotes neuronal adhesion and axonal extension	B, C	Potential long-term toxicity and poor in vivo clearance
Metal nanoparticle-based materials	Au, Pt, Ag nanoparticles	Extremely high (~10^4^–10^6^ S/cm)	Moderate; depends on surface modification	Generally nonbiodegradable	High modulus and poor mechanical match with soft neural tissues	Electrical signal amplification, electrochemical modulation, improved neural electrode interfaces	A, B	Risk of metal ion release, potential immunological reactions, long-term biosafety concerns
Phosphorus-based 2-dimensional nanomaterials	Black phosphorus (BP), phosphorene derivatives	Moderate to high; tunable bandgap (0.3–2.0 eV)	High biocompatibility	Biodegradable (degrades into phosphate ions)	Relatively flexible; suitable for composite scaffolds	Promotes neural regeneration, antioxidative effects, modulates cellular behavior	B	Instability in physiological environments, susceptibility to oxidation, complex synthesis and storage requirements

#### Conductive polymers

CPs, owing to their tunable electrical conductivity, have been widely used in engineering fields such as capacitors, flexible electronic devices, and organic light-emitting diodes (OLEDs) prior to their application in SCI repair [121]. However, their application in the biomedical field remains limited. In their native form, CPs are typically nondegradable, exhibit insufficient biocompatibility, and possess relatively high mechanical rigidity, which does not fully match the low modulus, high water content, and dynamic mechanical characteristics of spinal cord tissue [10]. Recent studies have demonstrated that composite modification and structural optimization strategies can markedly improve the biocompatibility, biodegradability, and mechanical compatibility of CPs. For example, incorporating PPy, PANI, PT, and its derivative poly (3,4-ethylenedioxythiophene) (PEDOT) with natural polymers (such as gelatin, chitosan, and hyaluronic acid) or biodegradable synthetic polymers [such as poly(lactic-co-glycolic acid) (PLGA) and polycaprolactone (PCL) ] can maintain electrical conductivity while reducing elastic modulus, enhancing flexibility, and enabling a certain degree of controlled degradation. Such composite materials have been extended to applications including biosensors, controlled drug delivery systems, neural electrode interfaces, and tissue engineering for bone, cardiac, neural, and muscle tissues [9,122].

The application of CPs in SCI varies across different stages of injury. In the acute phase, their use mainly focuses on anti-inflammatory effects and protection of the BSCB. During the subacute phase, the emphasis shifts toward promoting axonal regeneration and inhibiting scar formation. In the chronic stage or in cases of severe spinal cord cavitation, CPs are more commonly utilized as integrated platforms that combine structural bridging scaffolds with electrical signal conduction [10,11]. However, different injury models, such as contusion, compression, and complete transection, impose distinct requirements on mechanical compatibility, conductivity stability, and degradation rate of the materials, highlighting the need for a more systematic comparative framework in future studies.

CPs offer several advantages for SCI repair. First, their stable electroactivity can improve electrical signal transmission pathways within the injured region. When combined with ES, CPs can enhance the uniform distribution of electric fields, thereby improving the spatial precision of regulatory effects on neurons and glial cells. Second, CPs possess strong multifunctional compositing capability. They can readily incorporate neurotrophic factors [such as BDNF and nerve growth factor (NGF)], therapeutic drugs, or cells to construct multifunctional scaffold systems with sustained or gradient release properties. Such systems are particularly suitable for subacute and chronic SCI stages, where long-term molecular support is required [10,11,123]. In addition, studies by Chaudry and Stewart have shown that culturing NSCs on PEDOT or PPy surfaces can increase their differentiation toward neuronal lineages, suggesting that CPs may also have advantages in regulating cell fate decisions [124–126].

Despite marked progress in recent years, the application of CPs in SCI repair still faces several challenges. First, under long-term implantation conditions, certain conductive monomers, oxidative by-products, or dopants may induce chronic inflammatory responses and potential cytotoxicity, thereby affecting tissue compatibility and the stability of the neural microenvironment [9,127]. Second, the fabrication processes of CPs are relatively complex, and precise control over doping levels, conductivity uniformity, and structural stability remains challenging. In addition, long-term implantation may lead to a decline in electrical conductivity or interface instability, which could compromise sustained electrophysiological modulation [128]. Furthermore, most existing studies are still limited to small-animal models, and systematic validation in large-animal models, as well as long-term follow-up data, remain lacking. Consequently, the evidence base for clinical safety and functional stability is still relatively limited [129]. With the continued development of tissue engineering and bioelectronics, CPs show marked promise for SCI repair due to their unique electroactive properties. Through multidisciplinary integration of materials science, biology, and microelectronic technologies, it may be possible to optimize material performance while overcoming inherent limitations, thereby facilitating the practical application of CPs in neural repair and related biomedical fields [11,116].

#### Carbon-based materials

Carbon-based materials, which combine excellent electrical conductivity (10^3^ to 10^4^ S/cm) with flexibility, have been widely applied in cutting-edge technologies and industrial applications. For instance, they are commonly employed in energy storage devices such as ion batteries and supercapacitors, and in the electronics field for fabricating flexible electronic devices and transparent electrodes [9,130]. In recent years, carbon-based materials have attracted increasing attention in SCI repair due to their multidimensional advantages in promoting neural regeneration. Common carbon-based materials used for SCI repair include graphene, CNTs, carbon quantum dots (CQDs), and carbon black. Functionalized forms of graphene and CNTs, such as PEGylated or carboxylated derivatives, have been shown to possess good biocompatibility and low immunogenicity, helping to mitigate post-implantation inflammatory responses. This effect is attributed to surface modifications that improve dispersion and biointerface compatibility, reducing inflammatory reactions and making them suitable for SCI repair [131,132].

Studies indicate that carbon-based nanomaterials like CNTs and graphene not only promote neuronal adhesion, growth, and differentiation of NSCs but also serve as efficient platforms for ES therapy. Their excellent conductivity can mimic the electrical signaling environment of the nervous system, enhancing electric field transmission within the injury site and supporting neural regeneration [15,133]. Mechanically, their high strength and flexibility enable the construction of stable 3D scaffolds, providing physical support to cavitated or severed regions. Additionally, their large specific surface area facilitates the loading of neurotrophic factors, anti-inflammatory drugs, or stem cells, thereby enhancing overall reparative capacity [133,134]. Accordingly, carbon-based materials can not only promote axonal extension and NSC differentiation toward neurons but also synergistically enhance the effects of ES by improving the local electrical microenvironment, supporting neural network remodeling. Their structural support also helps maintain tissue integrity in the injury region, potentially benefiting BSCB stability and neural conduction recovery during the subacute and chronic phases [135]. Therefore, carbon-based materials are particularly suitable for subacute and chronic injuries, especially in contusion or transection models with marked tissue loss or cavitation, where they serve as conductive bridging scaffolds with both structural and electrical functions. However, direct implantation during the acute phase of peak inflammation requires careful evaluation of their effects on inflammatory responses.

However, unmodified or impurity-containing carbon materials may elicit potential cytotoxic effects, such as apoptosis, oxidative stress, or inflammatory reactions [136]. Another major challenge lies in their poor degradability: Carbon-based scaffolds are difficult to degrade in vivo and may persist long-term, raising safety concerns. Reports indicate that the degradability of CNTs and graphene is strongly dependent on their structural and surface properties [137]. Moreover, the in vivo distribution and long-term safety profiles of these materials remain insufficiently understood, with their biological behavior varying markedly depending on type, morphology, and functionalization, thereby hindering clinical translation [138,139]. Notably, certain modified CNTs can persist in vivo without inducing acute toxicity. For instance, Yang et al. [140] demonstrated that intravenously injected PEGylated graphene did not induce obvious toxicity in mice over a 3-month period, as evidenced by hematological, biochemical, and histological analyses; instead, the material accumulated in the liver and spleen and was gradually cleared. Despite these challenges, future strategies incorporating hydrogels as scaffolds and combining carbon-based materials with bioactive factors to construct conductive composite systems hold great promise for SCI therapy. Introducing CPs or engineering carbon-based composite hydrogel scaffolds may preserve their excellent conductivity while simultaneously improving toxicity and degradability issues. Such functionalized and rationally designed carbon-based composites not only provide stable physical support and a favorable electrical microenvironment in the injury site but also synergistically promote neuroprotection and functional recovery, offering safer and more effective therapeutic solutions for SCI repair.

#### Metal particle-based materials

Metal particle-based materials demonstrate unique potential in SCI repair. Typically incorporated into hydrogels or 3D scaffolds at the nano- or microscale, these particles enhance the electrical conductivity, bioactivity, and neuroregenerative performance of the composite materials. Metallic particles that have been studied for SCI repair include AuNPs, AgNPs, PtNPs, magnesium (Mg), zinc (Zn), and metal oxides such as iron oxide [141]. The primary advantage of metal particle-based materials in SCI repair lies in their intrinsic high electrical conductivity. This property not only provides a marked advantage for constructing highly conductive composite systems but also effectively enhances the transmission efficiency of ES signals and optimizes local electric field distribution. Consequently, metal particle-based materials are highly attractive for the fabrication of various conductive composites [142]. These materials also support multiple potential mechanisms in SCI repair. First, owing to their intrinsic high electrical conductivity, metal particles are highly attractive for the fabrication of conductive composites. By simulating the electrical microenvironment of neural tissue, they can facilitate axonal regeneration and functional recovery, particularly when combined with ES therapy. For example, an injectable Fe^3+^-coordinated double-network conductive hydrogel has been shown to reduce glial scar formation, promote remyelination, and provide neuroprotection [143]. Second, certain metal particles (e.g., Zn and Mg) can slowly release bioactive ions that exert neurotrophic, antioxidant, and immunomodulatory effects, thereby improving the local microenvironment and alleviating secondary inflammation and apoptosis. Liu et al. [144] designed hybrid microspheres with a Mg^2+^-crosslinked shell and Zn^2+^-loaded core to achieve temporally controlled ion release: early Mg^2+^ release to mitigate inflammation, followed by late Zn^2+^ release to promote neuroregeneration. Furthermore, conductive composites based on AuNPs, AgNPs, PtNPs, and iron oxide have been investigated for biomedical applications [12].

The advantages of metal particle-based materials in SCI repair are more specific to particular injury stages and pathological types. First, during the acute or subacute phases of SCI, when inflammation is active and oxidative stress is elevated, certain metal particle systems capable of releasing bioactive ions (e.g., Mg^2+^) can achieve anti-inflammatory regulation and microenvironment optimization through staged ion release, making them particularly suitable for early intervention strategies. Second, in contusion or compression injury models where the tissue structure is relatively intact but function is impaired, metal particle-based composites can promote functional recovery through electrical enhancement and ion-mediated modulation without relying on large-scale structural reconstruction. Third, in complete transection or severe cavitation models, metal particle-based materials can be combined with hydrogels or 3D porous scaffolds to construct conductive support systems, providing necessary structural stability and cell-adhesive interfaces to enable coordinated structural and functional repair [142,145,146].

Despite these advantages, metal particle-based materials also present marked challenges. The most prominent concern is potential cytotoxicity. Accumulation or excessive ion release in vivo can trigger oxidative stress, mitochondrial damage, and even neuronal death. For instance, AuNPs have been reported to exacerbate oxidative stress in the brain, reduce glutathione peroxidase (GPx) activity, and induce DNA damage and the up-regulation of inflammatory markers such as caspase-3 and Hsp70 [147]. Another limitation is their tendency to aggregate, which hinders uniform dispersion within composites and compromises electrical and mechanical consistency [148,149]. Moreover, knowledge of their long-term metabolism, safety, and immune responses remains incomplete. Prolonged exposure to certain iron oxide nanoparticles, for example, has been associated with hepatic lipid imbalance, inflammation, and increased cardiovascular risk [150]. We propose that future research should focus on improving biocompatibility through surface functionalization and antioxidant modification, developing controllable release systems to enable stage-specific ionic regulation, optimizing particle size and dispersion strategies to enhance material uniformity, and establishing systematic evaluation frameworks tailored to different injury stages and injury types. Such efforts will help identify the optimal therapeutic window for their application in the acute, subacute, and chronic phases of SCI, thereby facilitating their steady progression toward clinical translation.

#### Phosphorus-based 2D nanomaterials

Phosphorus-based 2D nanomaterials, such as BP and its derivatives, have attracted growing interest due to their unique physicochemical properties, with applications spanning optoelectronic devices, energy storage and conversion, and sensing. More recently, these materials have emerged as promising candidates for conductive biomaterials in SCI repair. BP exhibits excellent electrical conductivity and high carrier mobility, enabling efficient transmission of bioelectrical signals, modulation of membrane potentials, and regulation of signaling pathways to support axonal regeneration and remyelination. For instance, Liu et al. [151] developed BP@Hydrogel that, under a rotating magnetic field, generated stable electrical signals to activate the PI3K/Akt pathway, thereby promoting NSC differentiation into neurons and enhancing both behavioral and electrophysiological recovery. In addition, phosphorus-based 2D nanomaterials combine favorable biocompatibility with degradability. In vivo, they gradually degrade into nontoxic phosphate ions that can be metabolized by the body. This feature not only avoids long-term retention-associated risks but also contributes bioactive phosphate ions that participate in cellular metabolism and signaling, promoting neuronal differentiation and neurite outgrowth [152].

Based on the biological characteristics of phosphorus-based 2D nanomaterials, they are more suitable for SCI models in the subacute phase and the chronic reconstruction stage. During the subacute phase, they can exert combined reparative effects by modulating inflammation and promoting neural differentiation. In the chronic stage, particularly in injury models with partial tissue continuity, conductive composite hydrogels or 3D scaffolds incorporating these materials can enhance electrical signal transmission and support orderly axonal extension. However, their direct application during the peak of the acute inflammatory phase should be approached with caution, as their potential influence on oxidative stress levels requires careful evaluation. In addition, BP undergoes rapid oxidative degradation in humid environments, which can lead to the loss of conductivity and structural integrity, thereby limiting its capacity for long-term support [153]. Moreover, in complete transection or severe cavitation models, these materials are typically combined with structural scaffolds to provide the necessary physical support.

Nevertheless, their practical application remains limited by several inherent drawbacks. In particular, ROS generated during degradation may induce oxidative stress and trigger inflammatory responses, thereby raising potential safety concerns [154]. To address these issues, BP is often incorporated into conductive hydrogels, scaffolds, or composite systems to improve its stability and functionality. When combined with stem cells or neurotrophic factors, these composites can provide structural support, a conductive microenvironment, and biological signaling cues that synergistically enhance neuroprotection and functional recovery [155]. For example, BP incorporated into laminin-coated (LAMC) hydrogels and loaded with therapeutic agents markedly promoted angiogenesis and neural regeneration at SCI sites [156]. Taken together, phosphorus-based 2D nanomaterials, with their electrical conductivity, biocompatibility, degradability, and multifunctional regulatory properties, represent a cutting-edge strategy for SCI repair, offering both structural support and functional modulation.

From the perspective of overall material systems, different types of conductive biomaterials exhibit distinct applications in SCI repair. CPs possess excellent chemical modifiability and interfacial control capabilities. Their conductivity, surface charge, and bioactive molecule release can be tuned through doping or copolymerization, making them particularly suitable for constructing degradable conductive scaffolds, drug delivery systems, and cell carriers. However, their intrinsic conductivity is lower than that of carbon-based materials, and long-term implantation may pose risks of chronic inflammation due to oxidative by-products or dopants [11,121]. Carbon-based materials offer high intrinsic conductivity and superior mechanical strength, providing marked advantages in electrical signal transmission and structural stability. They are especially suitable for scaffolds addressing large chronic cavity lesions. Nevertheless, their poor biodegradability, potential cytotoxicity, and long-term metabolic concerns require further evaluation [136,157]. Metal particle-based materials (e.g., Au and Pt nanoparticles) are known for high conductivity and excellent electrocatalytic performance, enabling precise electrophysiological modulation and signal amplification. They can also be readily functionalized to deliver neurotrophic factors. However, their high hardness and elastic modulus make mechanical matching with the low-modulus spinal cord tissue challenging, and long-term implantation may induce metal ion release and immune responses [146]. Phosphorus-based 2D nanomaterials (e.g., BP and its derivatives) combine high conductivity with biodegradability. Their large 2D surface area enhances material–cell interface coupling, and they offer potential advantages in directional electrical signal conduction and local microenvironment regulation. These features make them promising for constructing oriented conductive scaffolds and promoting localized cell repair. Nevertheless, their oxidation sensitivity and complex fabrication remain limiting factors [153].

From the perspective of injury stages, the application focus of different materials also varies. During the acute phase, emphasis is placed on anti-inflammatory effects and protection of the BSCB; the subacute phase focuses on axonal regeneration, cell migration, and microenvironment optimization, whereas the chronic phase or severe cavitary injuries rely more on 3D scaffolds that provide structural support, directional conductivity, and long-term stability [158]. CPs primarily exert anti-inflammatory effects and maintain BSCB stability during the acute phase. In the subacute phase, they emphasize promoting axonal regeneration, inhibiting scar formation, and optimizing the local microenvironment. In chronic or severely cavitary injuries, CPs rely on their combined function as structural bridging scaffolds and electrical signal-conducting platforms to preserve tissue integrity and support neural functional recovery [121]. Carbon-based materials, due to their high conductivity and excellent mechanical strength, are better suited for the subacute and chronic stages, particularly in contusion or complete transection models with marked tissue loss or cavitation [135]. They can serve as conductive bridging scaffolds, providing both structural support and electrical modulation, although direct implantation during the acute inflammatory peak should be approached with caution. Metal particle-based materials derive much of their advantage from stage-specific ion release. For example, during the acute or subacute phase, certain metal particle systems capable of releasing Mg^2+^, Zn^2+^, or similar ions can modulate inflammation and oxidative stress, optimizing the microenvironment. This makes them particularly suitable for early intervention strategies [9,116]. Phosphorus-based 2D nanomaterials (e.g., BP and its derivatives) perform prominently during subacute and chronic reconstruction stages. In the subacute phase, they can exert combined reparative effects by modulating inflammation and promoting NSC differentiation. In the chronic phase, especially in models with partial tissue discontinuity, conductive composite hydrogels or 3D scaffolds can enhance electrical signal conduction and guide orderly axonal extension. However, their direct application during the acute inflammatory peak should also be approached cautiously to avoid adverse effects on oxidative stress levels [9,156].

### Mechanisms of conductive biomaterials in SCI repair

In recent years, the advent of conductive biomaterials has broken through longstanding bottlenecks in nervous system regeneration, bringing new therapeutic prospects for SCI patients. These materials exhibit favorable biocompatibility and mechanical properties while mimicking the intrinsic electrophysiological features of neural tissue, thereby creating an electrically conductive microenvironment that closely resembles physiological conditions [12]. When combined with ES, conductive biomaterials generate synergistic effects: The materials serve as “bridges” for electrical signal transmission, whereas ES acts as the “activation switch”. Together, they not only promote neuronal survival and axonal extension but also modulate the immune microenvironment and facilitate neural network reconstruction. This synergistic strategy holds promise to overcome multiple critical barriers in CNS regeneration [9]. The following section provides a systematic overview of the recent advances and potential mechanisms of conductive biomaterials in SCI repair, and presents a summary of the specific mechanisms of relevant conductive biomaterials over the past 5 years (Table 2).

**Table 2.** Summary of conductive biomaterial types and their mechanisms of action

Material types	Conductive components	Polymer materials	Bioactive components	Conductivity	Evidence level	Primary biological effects	Ref.
Conductive polymers	PEDOT:LS	GelMA/HAMA	/	5.1 × 10^−3^ S/cm	In vivo	Promotes NSC differentiation and neural regeneration	[115]
Conductive polymers	PPy	Gelatin	bFGF/TA/TIMP	3.75 × 10^−4^ S/cm	In vivo	Promotes angiogenesis, axonal regeneration, and microenvironment improvement	[195]
Conductive polymers	PPy/BaTiO3	SF	PER	7.3 × 10^−2^ S/cm	In vivo	Alleviates inflammation and excitotoxicity and promotes neuronal regeneration	[224]
Conductive polymers	PPy	Agar	TMP	7.66 × 10^−2^ S/cm	In vivo	Inhibits ferroptosis and promotes axonal regeneration	[225]
Conductive polymers	PANI	HA/Gel	His	2.0 × 10^−2^ S/cm	In vitro	Promotes neural cell proliferation and spinal cord regeneration	[226]
Conductive polymers	PPy	GelMA	TMP	1.49 × 10^−3^ S/cm	In vivo	Preserves BSCB integrity, inhibits oxidative stress, and promotes axonal regeneration	[202]
Conductive polymers	Aniline	Gelatin/OHA	NSCs	2.28 × 10^−2^ S/cm	In vitro/in vivo	Promotes neuronal differentiation, inhibits glial scar formation, and enhances spinal cord regeneration	[7]
Conductive polymers	PEDOT	TA	CSMA	1.61 × 10^−2^ S/cm	In vivo	Promotes neuronal differentiation and inhibits glial scar formation	[227]
Conductive polymers	PPy	Gel	BOC	4.49 × 10^−3^ S/cm	In vivo	Promotes neuronal and oligodendrocyte differentiation, inhibits glial scar formation, and enhances myelinated axon regeneration	[228]
Conductive polymers	PPy	Collagen/HA	/	1.89 × 10^−3^ S/cm	In vitro/in vivo	Protects BMSCs from ROS, promotes neuronal differentiation, and enhances neural regeneration	[164]
Conductive polymers	PANI	Gel	/	Not directly measured	In vivo	Restores spinal cord electrical signal conduction, reduces inflammation, and protects neurons	[229]
Conductive polymers	PPy	Gel/HA	/	4.3 × 10^−6^ S/cm	In vitro	Promotes NSC attachment and proliferation and supports neural tissue repair	[230]
Conductive polymers	PEDOT NPs	Gelatin/HA	/	8.3 × 10^−4^ S/cm	In vivo	Reduces inflammation and glial scar formation and promotes spinal cord regeneration	[128]
Conductive polymers	PPy	PVP	TA	1.8 × 10^−3^ S/cm	In vivo	Suppresses TNF-α-mediated inflammation, induces immunomodulation, and promotes neural repair	[231]
Conductive polymers	PEDOT:PSS	Alginate	/	Not directly measured	In vitro	Promotes MSC survival and proliferation and activates the VEGF signaling pathway to enhance nerve regeneration	[232]
Conductive polymers	PPy	Collagen	/	1.76 × 10^−3^ S/cm	In vitro	Promotes NSC differentiation, inhibits astrocyte differentiation, and supports nerve regeneration	[167]
Carbon-based materials	rGO/Fe_3_O_4_	GelMA/dECM	/	2.473 × 10^−2^ S/cm	In vivo	Promotes cell engraftment and axonal regeneration and enhances functional recovery	[233]
Carbon-based materials	MXene	GelMA	NSCs	3.6 × 10^−6^ S/cm	In vitro/in vivo	Guides neuronal growth and promotes spinal cord regeneration	[168]
Carbon-based materials	MXene	CHIC	NSCs	8.6 × 10^−3^ S/cm	In vivo	Promotes neuronal differentiation, axonal growth, and myelin regeneration	[6]
Carbon-based materials	rGO	XG	/	2.07 × 10^−2^ S/cm	In vivo	Promotes neural regeneration and inhibits glial scar formation	[234]
Carbon-based materials	CNT	GelMA	/	1.57 × 10^−2^ S/cm	In vitro/in vivo	Promotes neuronal proliferation and differentiation, modulates inflammation, and enhances axonal and myelin regeneration	[175]
Carbon-based materials	rGO	Shell	PDA	1.92 × 10^−3^ S/cm	In vivo	Alleviates inflammation, promotes angiogenesis, and supports axonal regeneration	[235]
Carbon-based materials	MXenes	PA/PVP	/	2.6 × 10^−3^ S/cm	In vivo	Promotes angiogenesis and axonal and myelin regeneration	[236]
Carbon-based materials	GO	PVA	MoS_2_	2.2 × 10^−3^ S/cm	In vitro/in vivo	Promotes neuronal differentiation and inhibits glial activation	[157]
Carbon-based materials	GO	Chitosan	/	5.4 × 10^−4^ S/cm	In vivo	Promotes neural cell adhesion and migration	[134]
Carbon-based materials	Melanin	PHB	/	8.48 × 10^−1^ S/cm	In vitro	Promotes DRG and motor neuron adhesion and growth	[237]
Metal particle-based materials	MoS_2_	GelMA/oxidized dextran	Wnt5a	2.98 × 10^−4^ S/cm	In vitro/in vivo	Alleviates neuroinflammation, enhances neuronal differentiation, and facilitates axonal regeneration	[183]
Metal particle-based materials	IL	SFMA	/	2.152 × 10^−3^ S/cm	In vivo	Promotes neural signal transmission and cell adhesion	[117]
Metal particle-based materials	Fe_3_O_4_	Guar Gum	Borax powder (1 wt %)	Not directly measured	In vitro/in vivo	Alleviates inflammation and promotes NSC differentiation	[238]
Metal particle-based materials	AuNPs	PCL/PLGA	BDNF/NGF/IKVAV	Not directly measured	In vivo	Promotes axonal guidance and spinal cord regeneration	[239]
Phosphorus-based 2D nanomaterials	GeP	HA/PDA	DA	3.65 × 10^−3^ S/cm	In vitro/in vivo	Promotes NSC differentiation and induces immunomodulation and angiogenesis	[240]
Phosphorus-based 2D nanomaterials	GeP_3_	HA	/	1.5 × 10^−3^ S/cm	In vitro/in vivo	Promotes NSC proliferation and migration and enhances vascularization and axonal and myelin regeneration	[241]
Phosphorus-based 2D nanomaterials	BP	CS/Gel	PDA	2.89 × 10^−3^ S/cm	In vitro/in vivo	Promotes NSC proliferation and differentiation and enhances axonal and myelin regeneration	[242]

#### Promotion of neural stem/progenitor cell differentiation into neurons

Neural stem/progenitor cells (NSCs/NPCs) represent a promising therapeutic strategy for a variety of neurological disorders. The ability to precisely control their development, self-renewal, and differentiation provides new opportunities for neuronal regeneration following SCI [159]. Recent studies have demonstrated that conductive biomaterials can effectively mimic the endogenous conductive microenvironment and markedly promote the lineage-specific differentiation of NSCs/NPCs into neurons, offering a compelling intervention strategy for neural regeneration [160] (Fig. S1). Some conductive components themselves have been shown to directly promote neuronal differentiation. For instance, **Park et al.** [161] reported that NSCs differentiated on graphene substrates exhibited the highest expression levels of neuronal markers (e.g., Tuj1/βIII-tubulin), which were markedly higher compared with nonconductive substrates. Further transcriptomic analysis by Tang et al. [162] revealed that graphene promoted neuronal differentiation by up-regulating the expression of multiple motor protein genes in NSCs (e.g., Cfap44, Dnah5, Dnah11, and Ccdc108), thereby driving lineage-specific fate commitment. In another study, **Li et al.** [163] developed a magneto-electric BP@Hydrogel system that generated stable electrical signals under a rotating magnetic field, effectively inducing neuronal differentiation of NSCs (Fig. S1B), with mechanistic involvement of the PI3K/AKT pathway. Moreover, Gao et al. [115] fabricated 3D-printed conductive biomimetic scaffolds encapsulating NSCs, which not only maintained high cell viability but also promoted neuronal differentiation markedly more than nonconductive scaffolds (Fig. S1C). ES itself can up-regulate neurotrophic factors, particularly BDNF [72,73], which activates the BDNF/TrkB–PI3K/Akt signaling axis [76]. The integration of conductive biomaterials enhances local electric field distribution, increases the precision of ES, and further augments the activity of signaling pathways such as PI3K/Akt, ultimately up-regulating genes associated with the cell cycle and differentiation (e.g., neuronal markers) to promote neuronal lineage specification. Additionally, Wu et al. [164] and Gao et al. [115] demonstrated that conductive biomaterials combined with ES could activate MAPK/ERK and Wnt/β-catenin pathways, which further facilitated neuronal differentiation (Fig. S1D). ES can also inhibit the Janus kinase (JAK)/signal transducer and activator of transcription 3 (STAT3) pathway, thereby reducing NSC differentiation into astrocytes (GFAP^+^) [165]. Based on these findings, conductive biomaterials may serve as amplifiers of ES, further suppressing JAK/STAT3 activity, reducing astrocytic differentiation, and limiting glial scar formation post-SCI. Furthermore, alterations in calcium ion channel activity and intracellular calcium dynamics are also implicated in NSC differentiation. For example, NSCs cultured on conductive 3D Ti_3_C_2_T_x_ MXene substrates under ES exhibited stronger calcium oscillations, reflecting enhanced calcium signaling transmission and neuronal activity. Periodic intracellular calcium fluctuations are critical regulators of neuronal development and plasticity, providing further support for neuronal differentiation [166]. Collectively, these findings suggest that conductive biomaterials, when combined with ES, can synergistically regulate intracellular signaling pathways, gene expression, and calcium dynamics to drive neuronal differentiation.

#### Promotion of axonal growth and neuronal adhesion

Conductive biomaterials also show marked promise in promoting neuronal adhesion, migration, and axonal outgrowth (Fig. S2). Their surfaces provide favorable physicochemical cues, where modulation of charge distribution, topography, and conductivity enhances NSC adhesion and spreading, leading to stable attachment within scaffolds. For example, Xu et al. [167] demonstrated that PPy-doped functional collagen hydrogels not only improved mechanical properties and biodegradability but also provided a suitable microenvironment that enhanced neuronal adhesion and proliferation. Additionally, tunable topographic structures (e.g., micro/nanoscale grooves, aligned fibers, or hierarchical patterns) incorporated into conductive biomaterials can facilitate spinal cord repair via multiple mechanisms. Grooved surfaces increase surface area, provide larger adhesion interfaces, and optimize ECM protein adsorption and receptor distribution. Such directional micro/nanostructures promote neuronal polarity and guide axonal extension, enabling organized neural network reconstruction and functional recovery. For instance, Cai et al. [168] showed that grooves of 10 to 50 μm achieved >80% axonal guidance efficiency in dorsal root ganglia (DRG). Similarly, other studies reported that biomimetic topographies improved cell alignment and axonal extension through cytoskeletal reorganization and mechanotransduction, as evidenced by Mobasseri et al. [169], who fabricated PCL/PLA scaffolds with microgrooves that enhanced axonal guidance and regeneration in nerve-bridge models. Conductive nanostructured films can further induce cytoskeletal remodeling through contact guidance, promoting neuronal differentiation, axonal elongation, and maturation [170].

Since the discovery of the RGD peptide motif as a mediator of cell adhesion, numerous materials have been functionalized with RGD to improve biocompatibility [171]. In SCI repair, synthetic materials incorporating collagen or gelatin expose RGD sequences that markedly enhance neuronal adhesion and migration [167]. We further propose that RGD may up-regulate growth-associated protein 43 (GAP-43) and tau expression, thereby facilitating axonal elongation. Moreover, RGD–integrin interactions can transmit mechanical signals to activate YAP/TAZ transcription factors, modulating the expression of genes associated with neuronal migration [172–174]. ES can further reinforce neuronal adhesion. For example, Yao et al. [175] reported that NSCs cultured on CNT/gelatin methacryloyl (GelMA) fibers aligned along the fiber axis, with adhesion markedly enhanced under ES (Fig. S2D). Mechanistically, ES activates voltage-gated calcium channels (VGCCs), inducing Ca^2+^ influx and triggering the Ca^2+^-calpain cascade, which promotes focal adhesion (FA) protein recruitment (talin, vinculin, paxillin) and focal adhesion kinase (FAK) phosphorylation at Tyr^397^. Activated FAK regulates Rho family guanosine triphosphatases (GTPases) (RhoA, Rac1, Cdc42), orchestrating actin cytoskeleton remodeling and growth cone dynamics, thereby directing axonal guidance and accelerating neuronal migration [176,177]. Although limited in number, existing studies support the role of ES in modulating intracellular Ca^2+^ levels to influence neurite guidance and adhesion [176]. In addition, conductive biomaterials may attenuate the inhibitory effects of myelin-associated molecules (Nogo-A, MAG, and OMgp), alleviating the hostile microenvironment that hinders axonal regeneration [178]. Conductive scaffolds can also provide electro-topographical cues to direct tubulin assembly, establish axonal polarity, and enhance lamellipodial dynamics in growth cones via Rac1-dependent actin polymerization, thereby promoting neurite initiation and branching [179,180]. Taken together, conductive biomaterials, in synergy with ES, can regulate the neuronal microenvironment, enhance adhesion and proliferation, and promote axonal guidance and extension, ultimately contributing to the reconstruction of functional neural networks after SCI.

#### Modulation of the immune and inflammatory microenvironment

Following SCI, the secondary injury cascade is accompanied by pronounced neuroinflammatory responses. Activated microglia and infiltrating macrophages release inflammatory mediators such as TNF-α, IL-1β, and ROS, which exacerbate apoptosis and tissue necrosis, ultimately worsening neurological dysfunction [31]. A growing body of evidence suggests that conductive biomaterials can modulate the immune and inflammatory microenvironment through diverse mechanisms, offering new strategies for tissue repair (Fig. S3). Certain conductive components themselves possess intrinsic immunomodulatory effects. For example, Di Mauro et al. [181] showed that graphene oxide nanosheets with small lateral dimensions markedly suppressed proinflammatory cytokine [TNF-α, IL-1β, and granulocyte-macrophage colony-stimulating factor (GM-CSF)]-induced Ca^2+^ signaling dysregulation and astrocyte hyperreactivity, demonstrating notable anti-inflammatory and neuroprotective properties. Similarly, Yan et al. [182] reported that graphene-based materials combined with ES modulated immune cell polarization, promoting the shift of macrophages from a proinflammatory M1 phenotype to an anti-inflammatory M2 phenotype, thereby validating the strong anti-inflammatory potential of graphene-based materials. Multifunctional composite conductive biomaterials designed to incorporate bioactive agents (e.g., drugs and cytokines) further enhance anti-inflammatory efficacy. Liu et al. [183] constructed a GOMW composite hydrogel composed of GelMA and oxidized dextran (Odex), doped with MoS₂ nanosheets and loaded with Wnt5a. Upon implantation into rat SCI sites, this hydrogel markedly down-regulated inflammatory cytokines (IL-6, TNF-α, and IL-1β) and enzymes involved in inflammatory responses [inducible nitric oxide synthase (iNOS), cyclooxygenase-2 (COX-2), and matrix metalloproteinases (MMPs)], thereby promoting repair (Fig. S3D). Likewise, Zhang et al. [184] developed Fe^3+^–polydopamine (PDA)–polyurethane–aniline trimer (PAT) conductive aligned nanofiber mats, which up-regulated COX5A and STAT6 while suppressing IL-1β, CD36, p-ERK, NF-κB, and NF-κB2 expression, thus promoting M2 macrophage polarization. This system also regulated intracellular signaling pathways such as Ca^2+^ and PI3K/Akt to enhance M2 polarization and inhibited NLRP3 inflammasome activation, highlighting the close involvement of NF-κB and STAT6 signaling modulation in attenuating neuroinflammation (Fig. S3E). Conductive biomaterials can also serve as carriers for anti-inflammatory compounds. For instance, **Wang et al.** [185] fabricated a biodegradable bilayer hydrogel membrane incorporating bazedoxifene (BZA), which gradually released BZA to modulate NF-κB activity and effectively suppress SCI-induced inflammation.

Our own findings further indicate that EES can partially suppress NLRP3 inflammasome activation, thereby exerting anti-inflammatory effects. We hypothesize that conductive biomaterials can amplify this effect through several synergistic mechanisms: (a) by improving electrical signal transmission and establishing a more stable and homogeneous local electric field, thereby enhancing the precision of cell regulation; (b) by modulating charge interactions at the material–cell interface, which influence immune cell adhesion, migration, and activation, facilitating M2 polarization and reducing cytokine release [175]; and (c) by leveraging mechano-electrical coupling effects that impact cytoskeletal remodeling and mechanosensitive pathways (e.g., FAK and RhoA/ROCK), thereby indirectly modulating immune responses [186–188]. Collectively, conductive biomaterials combined with ES can optimize the immune microenvironment by improving electrical signaling efficiency, regulating immune cell behavior, and orchestrating multiple signaling pathways, ultimately contributing to functional recovery after SCI.

#### Suppression of mitochondria-derived ROS and neuronal necroptosis

Neuronal loss following SCI is a major contributor to functional impairment, with mitochondrial dysfunction-induced ROS overproduction serving as a key trigger of necroptosis and secondary damage. Conductive biomaterials hold great potential in SCI repair by attenuating ROS-mediated neuronal necroptosis (Fig. S3). Numerous conductive biomaterials, including PPy, PANI, graphene, and CNTs, exhibit strong antioxidative regulatory properties [164,184]. Their conjugated electron systems enable electron transfer, redox reactions with ROS, and free radical scavenging, thereby mitigating oxidative stress [189]. Additionally, conductive biomaterials may regulate membrane potentials or activate stress-response pathways that promote nuclear translocation of nuclear factor erythroid 2-related factor 2 (Nrf2), a master regulator of antioxidant defenses. Nrf2 activation enhances the expression of downstream antioxidant proteins such as HO-1 and SOD2, which detoxify mitochondrial H₂O₂ and protect mitochondrial integrity [190,191]. Li et al. [192] demonstrated that Zn ions activated the Nrf2/HO-1 pathway while inhibiting NLRP3 inflammasome activation, thus facilitating motor function recovery after SCI. Similarly, Lin et al. [193] developed ZnO nanoparticle-loaded hydrogels, which up-regulated Nrf2 and HO-1 expression in SCI lesions, effectively lowering ROS levels and reducing neuronal apoptosis. ES itself has been shown to modulate neuroinflammation, and conductive biomaterials can provide uniform electrical interfaces to further optimize this effect, reducing superoxide anion generation. Moreover, conductive biomaterials can be functionalized with antioxidant bioactive agents. Natural polyphenols such as proanthocyanidins, quercetin, and resveratrol possess strong ROS-scavenging activity and inhibit NADPH (reduced form of nicotinamide adenine dinucleotide phosphate) oxidase. Liu et al. [194] engineered a tannic acid-doped hydrogel encapsulating mesenchymal stem cell-derived extracellular vesicles (MSCs-EVs), which effectively regulated ROS levels and promoted SCI tissue repair in vivo. In summary, conductive biomaterials mitigate mitochondrial ROS overproduction through multiple mechanisms, thereby reducing necroptosis and secondary injury severity. Their synergistic antioxidative, immunomodulatory, and bioactive delivery capabilities position them as powerful tools for protecting neurons and enhancing functional recovery after SCI.

#### Promotion of angiogenesis

During secondary injury after SCI, vascular rupture can induce microvascular spasms, leading to tissue ischemia, hypoxia, and vasogenic edema. Subsequently, endothelial cells become activated, adhesion molecule expression is up-regulated, and micro-thrombosis occurs, further exacerbating local microcirculatory disturbances. In the chronic phase of SCI, hypoxic conditions may stimulate angiogenesis; however, most newly formed vessels are immature, highly permeable, and structurally unstable, limiting effective perfusion and sustaining inflammation and tissue damage [32]. Furthermore, the expression of MMPs, particularly MMP-2 and MMP-9, is markedly elevated after SCI. These enzymes degrade the vascular basement membrane and ECM, resulting in BSCB disruption, increased vascular permeability, and aggravated inflammation (Fig. S4B) [195]. Conductive biomaterials have shown marked potential in promoting angiogenesis within the SCI microenvironment through multiple mechanisms, including regulation of cellular behaviors, activation of signaling pathways, and modulation of the local microenvironment (Fig. S4). For instance, ES applied through conductive matrices can activate signaling axes such as PI3K/Akt and MAPK/ERK to promote endothelial cell proliferation and neovascularization. Geng et al. [196] demonstrated that ES enhances endothelial cell secretion of fibroblast growth factor-2 (FGF2), which subsequently activates MAPK/ERK signaling and up-regulates VEGF expression. VEGF, through its receptor VEGFR2, activates endothelial nitric oxide synthase (eNOS), a key regulator of angiogenesis [197]. In parallel, Wei et al. [198] confirmed that direct current stimulation activates eNOS via the PI3K/Akt pathway, with phosphorylated eNOS directly enhancing enzymatic activity and nitric oxide (NO) production. NO release not only synergistically up-regulates VEGF expression but also enhances pro-angiogenic factors such as angiopoietin-1 and platelet-derived growth factor (PDGF) while simultaneously suppressing anti-angiogenic molecules such as endostatin [199]. Moreover, NO itself can activate PI3K/Akt and MAPK/ERK pathways, thereby promoting endothelial cell proliferation, migration, and lumen formation, establishing a positive feedback loop that supports effective vascular regeneration following SCI [200,201]. In addition, conductive biomaterials can indirectly facilitate angiogenesis by alleviating neuroinflammation and oxidative stress in the SCI region, thus creating a more favorable microenvironment for vascular repair. Multifunctional conductive composites doped with pro-angiogenic agents further enhance this effect. For example, Deng et al. [202] reported that ligustrazine-doped conductive hydrogels markedly up-regulated VEGF-A and VEGFR2 expression in SCI lesions, suggesting enhanced angiogenesis through VEGF-A/VEGFR2 signaling. Similarly, Fan et al. [195] developed an MMP-responsive conductive hydrogel loaded with basic fibroblast growth factor (bFGF) (Fig. S4C). Immunofluorescence analyses revealed a greater abundance of CD31^+^ capillaries in the treatment group, correlating with the increased expression of VEGF and other angiogenic factors (Fig. S4D).

#### Promotion of BSCB repair

Disruption of the BSCB is a critical driver of secondary SCI progression. Structural impairment of the barrier results in spinal cord edema, aggravated inflammation, and oxidative stress, ultimately leading to exacerbated neuronal and oligodendrocyte injury. Long-term BSCB dysfunction may further promote aberrant angiogenesis and glial scar formation, impairing the function of the neurovascular unit (NVU) and hindering spinal cord regeneration [178,203]. Importantly, increasing evidence demonstrates that conductive biomaterials can promote BSCB repair through multiple mechanisms (Fig. S4).

First, conductive scaffolds can reduce endothelial cell apoptosis following SCI while supporting endothelial migration and lumen formation to preserve vascular integrity. For example, Deng et al. [202] reported that conductive hydrogels decreased oxidative stress-induced endothelial injury and apoptosis, thereby enhancing vascular function. Immunohistochemical analyses further demonstrated increased expression of tight junction proteins (ZO-1 and Occludin) adjacent to the lesion, indicating BSCB repair (Fig. S4E). Similarly, Wang et al. [185] reported that composite hydrogels containing BZA effectively restored BSCB tight junction integrity when applied to SCI sites. Additionally, the conductive microenvironment provided by these biomaterials can inhibit MMP-9 activation, thereby reducing inflammatory damage to the barrier (Fig. S4C) [184]. Beyond barrier preservation, conductive biomaterials also promote angiogenesis and regulate the injury microenvironment, thereby improving BSCB function and perfusion, while simultaneously limiting astrocyte proliferation and glial scar formation. Reduced scar burden alleviates mechanical compression of the BSCB, decreases microvascular permeability, and creates a supportive niche for endothelial cell function and vascular stabilization [204]. Furthermore, as highlighted earlier, conductive biomaterials combined with ES can attenuate neuroinflammation by reducing immune cell infiltration and pro-inflammatory cytokine secretion, thus providing a favorable microenvironment for BSCB restoration.

Based on current research, SCI repair mediated by conductive biomaterials involves multi-level biological regulatory processes. Specifically, these mechanisms differ in terms of the target cell types, the pathological stages at which they act, and the associated molecular signaling pathways.

First, at the cellular level, different repair mechanisms primarily target cell populations that play key regulatory roles during post-injury regeneration. For example, in promoting directed neuronal differentiation, conductive biomaterials mainly act on NSCs/NPCs and their differentiation lineages; by regulating their cell fate, they determine the cellular composition of the regenerating tissue and effectively increase the neuronal pool [160,162]. Mechanisms that promote axonal regeneration and neuronal adhesion mainly target mature neurons and their growth cone structures, facilitating axonal extension and neural network reconstruction through modulation of cell–matrix interactions and cytoskeletal dynamics [179,180]. Immune microenvironment modulation mainly targets immune cells such as microglia and macrophages, improving the inflammatory milieu at the injury site by regulating their activation status and phenotypic polarization [181,182]. ROS scavenging and mitochondrial protection mechanisms primarily target damaged neurons and their mitochondria, reducing oxidative stress to prevent neuronal death and mitigate secondary injury [191]. Additionally, mechanisms involved in vascular regeneration and BSCB repair primarily act on endothelial cells and the NVU, promoting microvascular formation and maintaining vascular barrier integrity to provide stable blood supply and metabolic support for neural regeneration [178,195,203].

Second, regarding the temporal aspect, these mechanisms exhibit dynamic changes during SCI repair, with different processes dominating at distinct pathological stages. Immune and inflammatory regulation, as well as ROS scavenging, mainly occur from the acute to early subacute phase, alleviating secondary injury by suppressing inflammation and oxidative stress, thereby creating a favorable environment for subsequent tissue repair [30,205]. The promotion of NSC/NPC differentiation into neurons and axonal regeneration is concentrated in the subacute to early tissue reconstruction phase, when endogenous NSCs/NPCs are active and cellular plasticity is high, facilitating the formation of regenerative neural networks [206]. In contrast, angiogenesis and BSCB repair mainly participate in the mid-to-late tissue remodeling stage, restoring microcirculation and maintaining NVU stability, thereby providing sustained structural and metabolic support for neural tissue regeneration and long-term functional recovery [207,208].

Finally, at the level of molecular regulatory pathways, the signaling networks underlying different repair mechanisms exhibit distinct functional characteristics. In mechanisms promoting NSC/NPC differentiation, conductive biomaterials primarily engage neurogenesis-related pathways such as BDNF/TrkB–PI3K/Akt, MAPK/ERK, and Wnt/β-catenin. These pathways up-regulate the expression of neuron-specific genes and enhance cell survival and synapse formation. At the same time, inhibition of the JAK/STAT3 pathway reduces NSC differentiation into astrocytes, thereby limiting glial scar formation and optimizing the composition of regenerating cells [76,115,164,165]. In contrast, mechanisms associated with axonal regeneration largely depend on VGCC–Ca^2+^ signaling, FAK, and Rho family GTPase-mediated cytoskeletal regulation networks. These pathways promote neuronal migration and directed axon extension by modulating FA complex formation, actin reorganization, and growth cone dynamics [176,177,179,180]. In immune modulation, conductive biomaterials exert their effects mainly through inflammation-related pathways, including NF-κB, STAT6, and the NLRP3 inflammasome. These pathways regulate the release of inflammatory cytokines and macrophage polarization, suppressing pro-inflammatory responses while promoting tissue repair-associated anti-inflammatory phenotypes [183,184]. Mechanisms targeting oxidative stress and neuroprotection primarily rely on the Nrf2/HO-1 antioxidant axis and ROS scavenging responses, which up-regulate intracellular antioxidant enzymes, maintain mitochondrial homeostasis, and reduce free radical accumulation, thereby mitigating ROS-mediated neuronal damage [192,193]. Furthermore, vascular regeneration and BSCB repair mechanisms mainly involve PI3K/Akt, MAPK/ERK, and VEGF/VEGFR2–eNOS signaling pathways to promote endothelial cell proliferation, migration, and neovascularization, while enhancing endothelial function and microcirculatory stability, thus providing sustained metabolic and nutritional support to the injury site [195–197].

Overall, these repair mechanisms display functional specialization and stage-specific cooperation at the levels of cellular targets, temporal activity, and signaling pathways. In the early phase of injury, they primarily mitigate secondary damage by suppressing inflammation and oxidative stress. During the subacute phase, the focus shifts to promoting NSC differentiation, axonal regeneration, and neural network reconstruction. In the later stages, vascular regeneration and BSCB repair are critical for maintaining a stable regenerative microenvironment and supporting long-term functional recovery. By virtue of their unique electrophysiological properties and multifunctional regulatory capacity, conductive biomaterials can integrate these multi-level biological processes within a single regenerative microenvironment, producing a synergistic effect that enhances SCI repair.

## Future Perspectives

Conductive biomaterials can provide both electrical and structural support, overcoming the limitations of traditional nonconductive scaffolds such as conventional hydrogels and collagen, which cannot mimic the electrophysiological microenvironment of nerve tissue. This gives them marked advantages in SCI repair. With their strong conductivity, mechanical performance, and biocompatibility, conductive biomaterials not only reproduce the electrical features of neural tissue but also support neuronal adhesion, growth, and functional maintenance. Compared with materials that only offer structural support, conductive scaffolds use their electrical properties to improve signal transmission in the injury site, promote axon regeneration, enhance remyelination, and help rebuild neural circuits. The functional performance of conductive biomaterials depends not only on their macroscopic structure but also on molecular-level design. In recent years, researchers have increasingly employed molecular engineering strategies to finely tune CPs, aiming to achieve a synergistic optimization of conductivity, biocompatibility, and biological functionality [209,210]. From a materials design perspective, the properties of conductive biomaterials—particularly conjugated polymers—largely depend on the tunability of their molecular chemical structures. Key factors influencing conductivity, biocompatibility, and therapeutic activity include the polymer backbone, side-chain modifications, and functional group integration [121]. (a) Backbone structure: The π-conjugated system, composed of alternating single and double bonds (e.g., PPy, PANI, and PEDOT), determines the degree of electron delocalization and charge transport capability. By adjusting monomer type, conjugation length, and donor–acceptor structures, the carrier mobility of the polymer can be effectively modulated, thereby altering its conductivity and electrical signal transmission capacity [10,210]. (b) Side-chain modifications: Introducing hydrophilic or bioactive side chains (e.g., polyethylene glycol, carboxyl, or amino groups) can markedly improve water dispersibility, surface wettability, and cellular compatibility. Moreover, by modulating surface charge density and spatial conformation, these modifications enhance cell adhesion and protein adsorption [211]. (c) Side-chain effects on mechanical and degradation properties: Side-chain structures also influence polymer flexibility, degradability, and stability under physiological conditions, further optimizing their performance for neural tissue engineering applications [11,121]. (d) Functional group integration: Incorporating specific biofunctional groups or conjugating bioactive molecules (e.g., RGD peptides, antioxidant molecules, or growth factor-binding sites) onto the polymer backbone or side chains not only strengthens interactions with the ECM but also imparts additional biological functions, such as antioxidant, anti-inflammatory, or cell differentiation-promoting activities [11,212].

A range of in vitro and in vivo studies have shown that CP-based scaffolds, including PEDOT and PPy, as well as composites incorporating graphene or MXene, can promote NSC differentiation toward neurons, guide directional axon extension, and strengthen functional recovery [15,128,213]. In addition, certain metal-based conductive hydrogels show antioxidant and anti-inflammatory activity, which helps suppress secondary inflammation and reduce glial scar formation, further supporting tissue repair [214]. By combining electrical regulation with biochemical modulation, conductive biomaterials create a microenvironment closer to physiological conditions than traditional materials. These materials hold strong promise for promoting axon regeneration and functional recovery and represent a highly promising strategy for SCI repair. Although conductive biomaterials have demonstrated marked potential for promoting neural repair after SCI in experimental studies, their clinical application remains very limited, with several key translational barriers restricting their use in patients. First, material stability is a critical factor affecting clinical translation. Some CPs or nanocomposite scaffolds may undergo structural degradation or loss of conductivity within the complex physiological environment of the injured spinal cord. In particular, whether the integrity of the conductive network and its electrical signal transmission can be maintained under long-term implantation conditions remains to be systematically evaluated [10]. Therefore, achieving stable electrical performance while ensuring material degradability has become a central challenge in the design of conductive biomaterials [11]. Second, biological safety requires further comprehensive evaluation. Although most conductive biomaterials exhibit good biocompatibility in short-term experiments, certain conductive components (such as carbon-based nanomaterials or some conjugated polymers) may induce chronic inflammatory responses, immune activation, or glial scar formation following long-term implantation, negatively affecting the regenerative microenvironment [215,216]. Optimizing material composition, surface modification, and biocompatibility to minimize potential immune responses and improve long-term implantation safety remains an important focus for future research. Third, manufacturing and reproducibility also limit clinical translation. Many conductive biomaterials are still at the laboratory research stage, and their fabrication typically involves complex chemical polymerization, nanocomposite assembly, or multi-component functionalization processes. These complexities pose marked challenges for scalable production and strict quality control. The lack of standardized, reproducible manufacturing processes increases batch-to-batch variability and represents a major barrier to clinical application [217].

To overcome the aforementioned challenges and facilitate clinical application, future research should establish a more systematic translational pathway. First, at the material design level, conductive biomaterials should be optimized through rational engineering strategies to achieve balanced physicochemical properties, including conductivity, degradability, and mechanical characteristics matched to spinal cord tissue, while minimizing potential cytotoxicity and inflammatory responses [11,218]. Second, at the preclinical evaluation level, standardized assessment systems should be established to systematically evaluate the materials’ electrical performance, biocompatibility, neuroregenerative capacity, and long-term functional recovery in both in vitro and in vivo models, with rigorous validation in nonhuman primate models [210]. Third, integration with multimodal therapeutic strategies may further enhance efficacy. Conductive biomaterials can serve as multifunctional platforms combined with ES, stem cell transplantation, or neurotrophic factor delivery to synergistically modulate the injury microenvironment, thereby promoting axonal regeneration and neural functional recovery [123,218]. Finally, the development of scalable manufacturing technologies and regulatory pathways is critical for clinical translation. Establishing reproducible fabrication processes, ensuring batch-to-batch consistency, and conducting long-term safety and efficacy studies will help bridge the gap between basic research and clinical application. Through interdisciplinary collaboration across materials science, neurobiology, and clinical medicine, conductive biomaterials hold promise to advance from experimental platforms toward a viable therapeutic strategy for SCI [130,209].

Nevertheless, marked strides have already been made toward clinical application. For example, the NeuroRegen collagen scaffold, developed by Dai’s team, has demonstrated remarkable efficacy in both preclinical and clinical studies, serving as a representative example of successful translation from bench to bedside. In multiple rodent SCI models, NeuroRegen scaffolds facilitated orderly axonal regeneration, promoted remyelination, and markedly improved motor function [219]. In canine models, the scaffold supported functional reconnection of nerve fibers across the lesion site, leading to improved locomotor performance and neural conduction recovery [220]. In nonhuman primates (e.g., rhesus monkeys), when combined with engineered human spinal cord-like tissue transplantation, the scaffold facilitated the formation of integrated neural networks, reconstruction of sensory and motor pathways, and substantial functional recovery. Importantly, multicenter clinical trials involving over 100 patients with both acute and chronic complete SCI have been completed. Preliminary follow-up results indicated favorable safety outcomes and partial recovery of sensory, urinary, and motor functions in some patients, highlighting the clinical potential of this therapeutic strategy [221–223]. Building on these achievements, future research could incorporate conductive components into established scaffold systems such as NeuroRegen, creating biomaterials with both excellent biocompatibility and enhanced electrical regulation capacity, thereby improving therapeutic efficacy and accelerating clinical translation.

To drive clinical translation further, the design of conductive biomaterials must transcend simple electrical conduction and evolve into multifunctional platforms capable of intelligently modulating the local microenvironment. For example, conductive scaffolds could be engineered to co-deliver antioxidants, neurotrophic factors, or small-molecule drugs, enabling coordinated regulation of oxidative stress, inflammation, and angiogenesis. Moreover, the construction of stimuli-responsive release systems—triggered by physiological signals such as pH, ROS, or ES—would allow precise spatiotemporal delivery of therapeutic agents. Integration of conductive scaffolds with cutting-edge technologies such as stem cells, extracellular vesicles, or biosensors also opens opportunities to establish comprehensive “integrated therapeutic systems” that simultaneously support neural modulation, tissue regeneration, and functional restoration. With continuous advances in materials science and neuroengineering, conductive biomaterials combined with ES hold strong promise to achieve genuine clinical breakthroughs in the near future, offering SCI patients tangible functional improvement and new hope for recovery.

## Conclusion

Neural functional reconstruction after SCI remains a major challenge in the field of nerve regeneration. SCS has emerged as a key technological advance in neurorehabilitation due to its unique advantages in promoting spinal cord repair. However, with the deepening of research and clinical applications, the limitations of SCS have become increasingly apparent, particularly in cases of complete SCI or severe structural damage, where its restorative effects remain constrained. The advent of conductive biomaterials offers a promising strategy to overcome these bottlenecks. These materials not only provide a stable and efficient platform for electrical signal conduction but also create biomimetic electrical microenvironments and 3D scaffolds in the injury site, facilitating directed differentiation of NSCs, orderly axonal growth, myelin reconstruction, and angiogenesis. Moreover, they can mitigate secondary injury through immunomodulation, achieving multidimensional synergistic effects on neuroprotection and regeneration. Notably, when combined with ES, conductive biomaterials can markedly amplify the regulatory effects of electrical signals, advancing SCI repair toward a “precise and multimodal” paradigm. Although clinical translation still faces numerous challenges, representative outcomes—such as the NeuroRegen collagen scaffold—demonstrate encouraging prospects. Overall, the development of conductive biomaterials not only reveals novel mechanisms underlying SCI repair but also lays a critical foundation for the future implementation of personalized and intelligent therapeutic strategies.

## Data Availability

No data were used for the research described in the article.

## Supplementary Materials

Supplementary 1 Figs. S1 to S4 Reference [243]
