# Supplementary material for: Mechanisms and Applications of Conductive Biomaterials in Spinal Cord Injury Repair
Source: Biomater Res. 2026 Jun 11;30:0381. doi: 10.34133/bmr.0381 (PMC13254569; doi:10.34133/bmr.0381)
Supplement: Supplementary 1 — Figs. S1 to S4 Reference [243] [file bmr.0381.f1.docx]

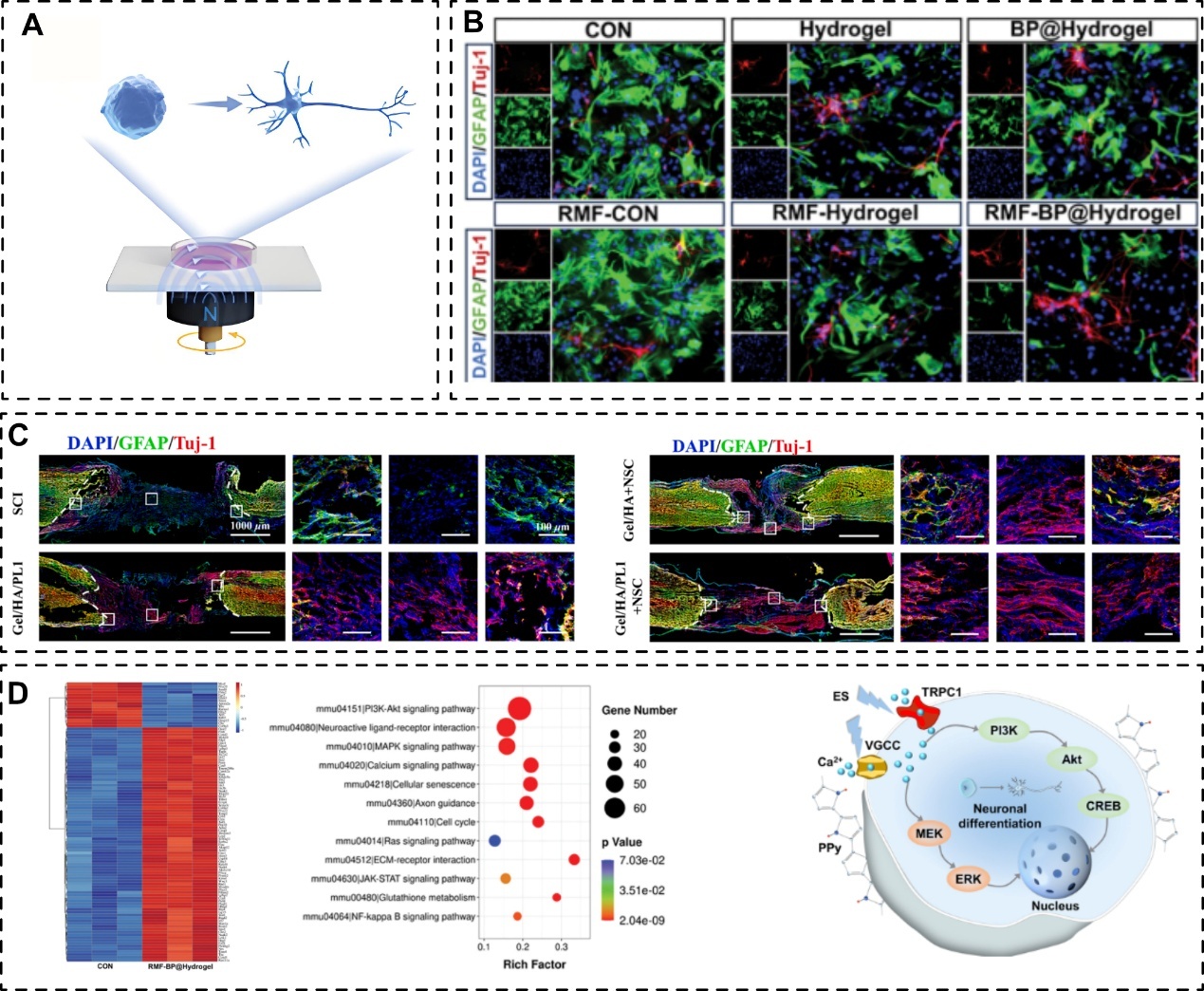


**Fig S1**. Conductive biomaterials promote NSCs differentiation into neurons. (A) Schematic showing that NSCs, under the combined action of conductive biomaterials and a rotating magnetic field, generate stable currents and are induced to differentiate into neurons; (B) Immunofluorescence staining of β-tubulin III (Tuj-1) and glial fibrillary acidic protein (GFAP) further analyzes the differentiation ratio of NSCs into neurons. Reproduced with permission[157]. Copyright, 2024, Wiley; (C) NSCs loaded onto conductive scaffolds also show a tendency toward neuronal differentiation, and immunofluorescence staining of Tuj-1 and GFAP in spinal cord longitudinal sections further confirms this result. Reproduced with permission[119]. Copyright, 2023, Elsevier; (D) Schematic model of the mechanisms by which conductive biomaterials promote NSC differentiation into neurons. Reproduced with permission[157, 173]. Copyright, 2024, Wiley; Copyright, 2021, American Chemical Society (ACS).


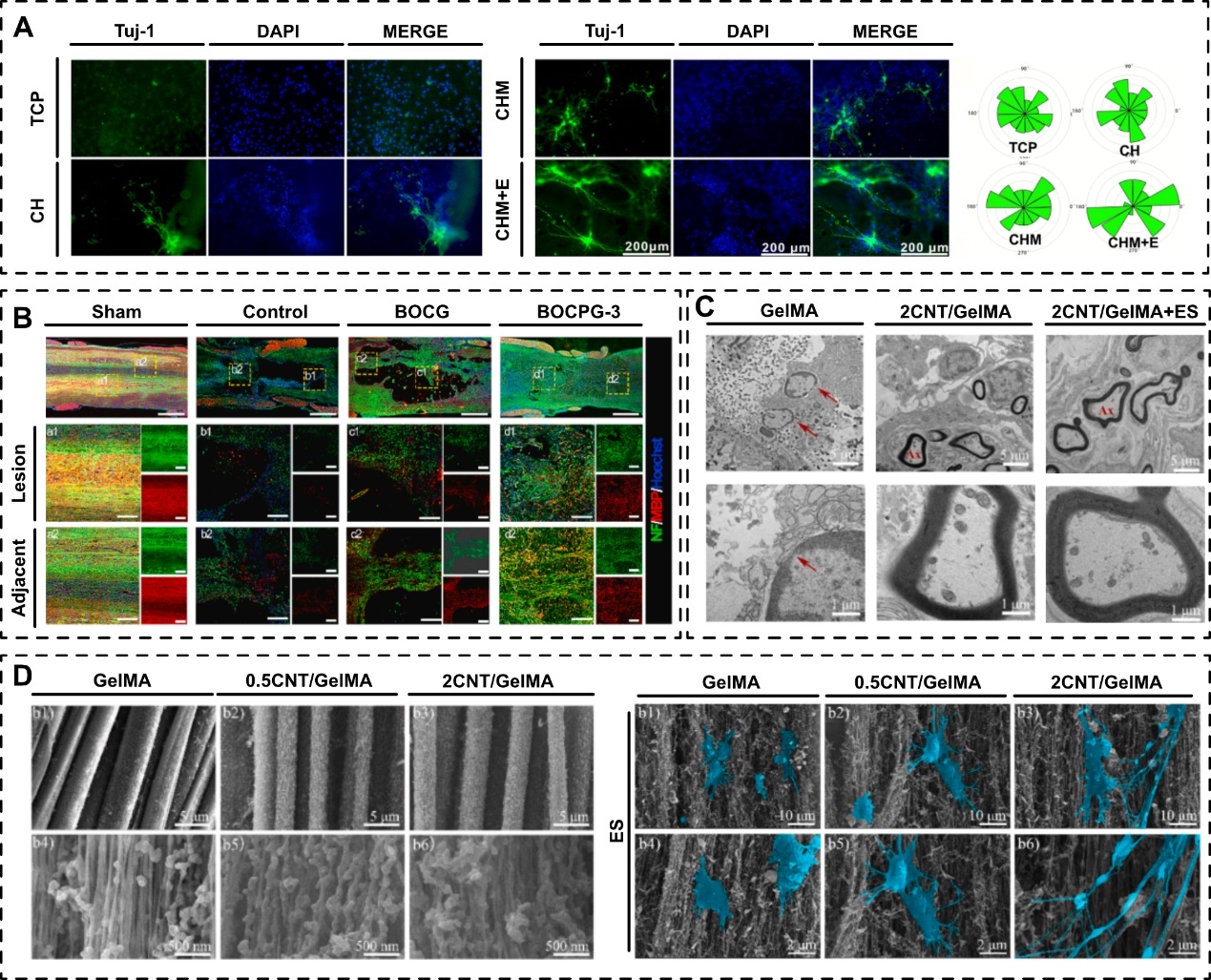


**Fig S2.** Conductive biomaterials promote axonal growth and neuronal adhesion. (A) NSCs cultured in conductive hydrogels (CHM) combined with electrical stimulation show significantly enhanced axonal growth, with longer axons and clearer orientation as shown by immunofluorescence and rose plots. Reproduced with permission[6]. Copyright 2023, Elsevier; (B) Borax-doped, chondroitin sulfate-modified polypyrrole (BOC-PPy) conductive hydrogel significantly promotes axonal regeneration, with immunofluorescence staining showing markedly increased density of myelin basic protein (MBP)-positive myelin sheaths. Reproduced with permission[172]. Copyright 2022, Elsevier; (C) Transmission electron microscopy (TEM) reveals that carbon nanotube (CNT)-doped hydrogels facilitate axonal repair, which is further enhanced by electrical stimulation; (D) Scanning electron microscopy (SEM) shows that different CNT concentrations do not significantly alter composite morphology, while GelMA doped with carbon-based composites exhibits stronger NSC adhesion. Reproduced with permission[182]. Copyright 2024, Elsevier.


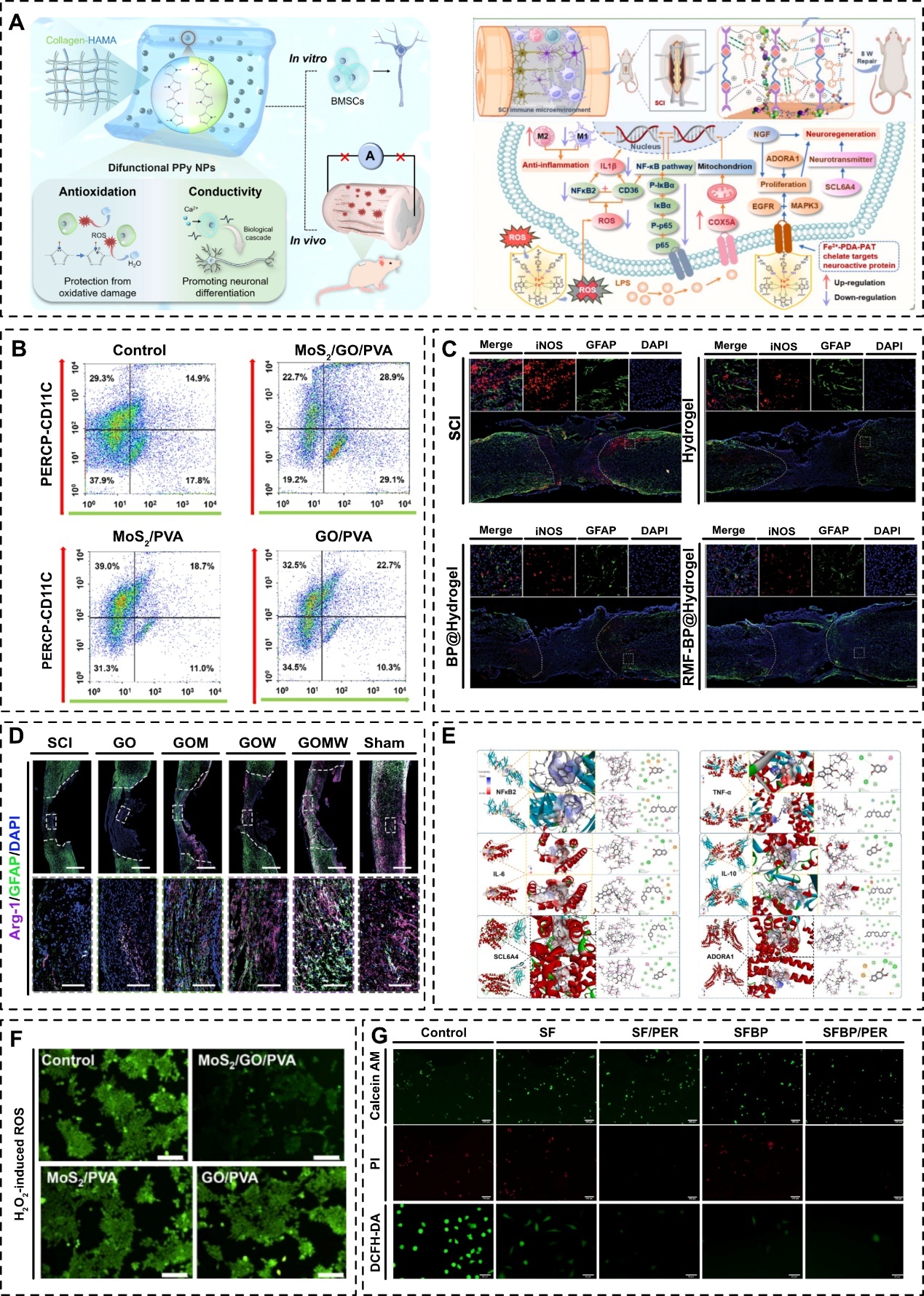


**Fig S3.** Conductive biomaterials modulate immune-inflammatory responses and suppress ROS-mediated oxidative stress. (A) Schematic illustration of inflammation regulation and ROS scavenging mediated by conductive biomaterials. Reproduced with permission[173, 210]. Copyright 2021, American Chemical Society; Copyright 2025, Elsevier; (B) Flow cytometry results confirm the effects of GO-doped conductive biomaterials on macrophage (RAW264.7) activity. Reproduced with permission[163]. Copyright 2022, BioMed Central (BMC); (C, D) Conductive biomaterials inhibit inducible nitric oxide synthase (iNOS) while upregulating arginase-1 (Arg-1) expression to regulate neuroinflammation. Reproduced with permission[186]. Copyright 2024, Wiley; (E) Molecular docking suggests that catechol fragments within bioinspired conductive oriented nanofiber felt chelates form multiple intermolecular interactions with inflammation-related proteins, effectively suppressing inflammatory responses. Reproduced with permission[210]. Copyright 2025, Elsevier; (F, G) ROS scavenging mediated by conductive biomaterials. Reproduced with permission[167, 186]. Copyright 2024, Wiley; Copyright 2024, Elsevier.


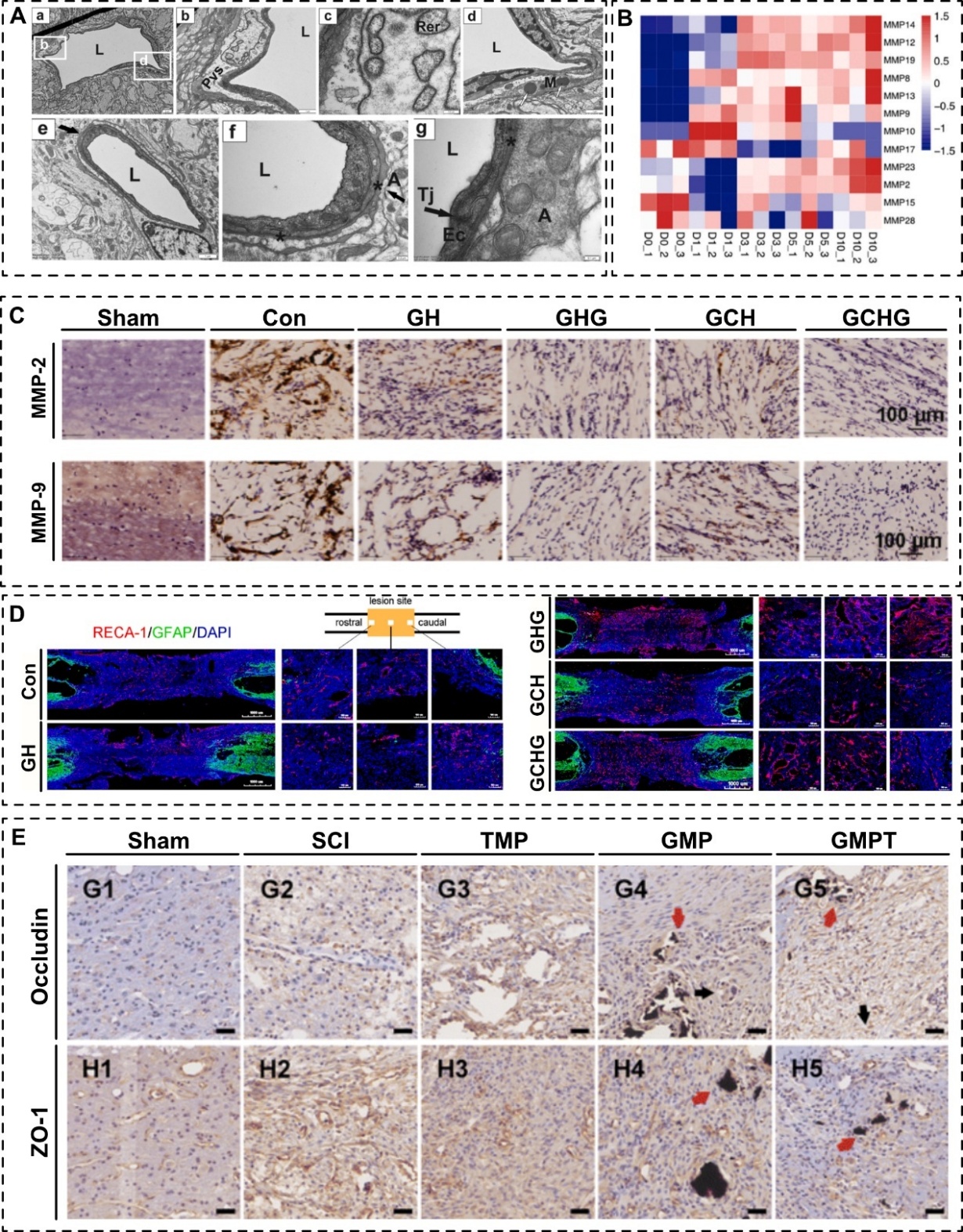


**Fig S4.** Conductive biomaterials promote angiogenesis and repair of the blood–spinal cord barrier (BSCB). (A) Transmission electron microscopy (TEM) image of perivascular ultrastructure in the spinal cord. Reproduced with permission[229] (A: astrocyte; Ec: endothelial cell; L: lumen; microglia; Rer: rough endoplasmic reticulum; Tj: tight junction). Copyright 2017, Springer; (B) After spinal cord injury (SCI), matrix metalloproteinase (MMP) gene expression is significantly upregulated in spinal cord tissue; (C, D) Synthetic MMP-responsive conductive hydrogels downregulate MMP-2 and MMP-9 protein levels and promote angiogenesis. Reproduced with permission[166]. Copyright 2022, Elsevier; (E) Conductive hydrogels doped with ligustrazine increase expression of tight junction proteins (ZO-1, Occludin) in the BSCB, indicating a reparative effect (scale bar: 40 μm). Reproduced with permission[170]. Copyright 2024, Royal Society of Chemistry (RSC).
